# Dual-Driven Hemostats Featured with Puncturing Erythrocytes for Severe Bleeding in Complex Wounds

**DOI:** 10.34133/2022/9762746

**Published:** 2022-05-31

**Authors:** Haoyu Qiu, Guangqian Lan, Weiwei Ding, Xinyu Wang, Wenyi Wang, Dahua Shou, Fei Lu, Enling Hu, Kun Yu, Songmin Shang, Ruiqi Xie

**Affiliations:** ^1^State Key Laboratory of Silkworm Genome Biology, College of Sericulture, Textile and Biomass Sciences, Southwest University, Chongqing 400715, China; ^2^Chongqing Engineering Research Center of Biomaterial Fiber and Modern Textile, Chongqing 400715, China; ^3^Division of Trauma and Surgical Intensive Care Unit, Research Institute of General Surgery, Jinling Hospital, Medical School of Nanjing University, Nanjing, 210002 Jiangsu Province, China; ^4^Institute of Textiles and Clothing, The Hong Kong Polytechnic University, Kowloon, Hong Kong

## Abstract

Achieving rapid hemostasis in complex and deep wounds with secluded hemorrhagic sites is still a challenge because of the difficulty in delivering hemostats to these sites. In this study, a Janus particle, SEC-Fe@CaT with dual-driven forces, bubble-driving, and magnetic field– (MF–) mediated driving, was prepared via *in situ* loading of Fe_3_O_4_ on a sunflower sporopollenin exine capsule (SEC), and followed by growth of flower-shaped CaCO_3_ clusters. The bubble-driving forces enabled SEC-Fe@CaT to self-diffuse in the blood to eliminate agglomeration, and the MF-mediated driving force facilitated the SEC-Fe@CaT countercurrent against blood to access deep bleeding sites in the wounds. During the movement in blood flow, the meteor hammer-like SEC from SEC-Fe@CaT can puncture red blood cells (RBCs) to release procoagulants, thus promoting activation of platelet and rapid hemostasis. Animal tests suggested that SEC-Fe@CaT stopped bleeding in as short as 30 and 45 s in femoral artery and liver hemorrhage models, respectively. In contrast, the similar commercial product Celox™ required approximately 70 s to stop the bleeding in both bleeding modes. This study demonstrates a new hemostat platform for rapid hemostasis in deep and complex wounds. It was the first attempt integrating geometric structure of sunflower pollen with dual-driven movement in hemostasis.

## 1. Introduction

Hemorrhage shows a high mortality rate of more than 30% and has been become a momentous issue in emergency scenarios during both civilian and military trauma [1, 2]. Hence, mitigating blood loss and controlling hemorrhage using hemostats is essential to ensure effective pre-hospital care for the wounded and patients with massive bleeding [3]. Although commercial hemostatic materials, such as chitosan-based Celox™ dressing [4], zeolite-based Combat Gauze® [5], and oxidized regenerated cellulose-based Surgical® hemostats [6], are available for massive bleeding control, they cannot achieve rapid hemostasis in complex wounds with deep and secluded hemorrhagic sites because it is difficult for them to reach secluded and deep sites, and thus blood coagulation occurs exclusively on superficial wounds [7, 8]. Therefore, effective and rapid hemostatic agents for deep and complex wounds with secluded hemorrhagic sites are needed.

To achieve effective and rapid hemostasis in complex wounds with deep and secluded hemorrhagic sites, as mentioned above, a hemostatic agent should first fully and extensively contact the bleeding sites [9], especially in irregular bleeding cavities with fine ravines and voids. To fully and extensively contact bleeding sites, shape-adaptive hemostatic foams have been developed with rapid volume expansion ability and fast liquid-absorbing capacity upon contact with blood [10, 11]. Efficient hemostasis, therefore, occurs rapidly inside the hemocoel when the shape of the hemostat adapts to wound cavities. However, the macroscopic form and limited flexibility of the foams inevitably prevent the foams from sufficiently contacting the bleeding sites in the fine ravines and voids of bleeding cavities. Insufficient adaptation to hemorrhagic hemocoels probably leaves part of the bleeding sites without external hemostatic treatment, thus prolonging the hemostasis time [2, 3]. To address the insufficient adaptation issue, powered hemostat systems with gas-propelling properties have been developed to facilitate better contact with bleeding sites, especially in irregular wounds [12, 13]. For example, a Janus particle system composed of microporous starch@CaCO_3_ can generate CO_2_ bubbles upon contacting blood with the synergistic use of protonated tranexamic acid (TXA-NH_3_^+^). During the bubble release, a propulsion force is generated to push the particles upstream. Because the propulsion force of the bubbles occur in various directions, each particle travels in random directions, and thus full contact is achieved between the hemostatic agent and bleeding sites [13]. Unfortunately, the solo gas-propelling force may be not lasting when CaCO_3_ was depleted gradually, failing to deliver particles to the bleeding sites at the bottom of deep severe wounds.

Therefore, to reach the deep bleeding sites, a hemostat with lasting driving force must be required. These materials should exhibit the ability to respond to external stimuli, such as magnetic fields (MFs), biological stimuli, ultrasound, temperature, light, and electricity [14, 15]. In particular, because of MF-responsive platforms' quick response, safe tissue penetration, and accurate positioning [16], important progress have been made toward developing MF-responsive platforms. For instance, we have previously developed magnetic microporous starch particles to treat bleeding in I-shaped and J-shaped irregular/deep wounds [17]. Similarly, magnetically controlled protein nanocontainers (NCs) have been developed to precisely deliver and accumulate hemostats at the bleeding site under an applied magnetic field to inhibit clot lysis [18]. Therefore, MF-mediated driving force should be good candidate for deep and secluded hemorrhagic sites.

In addition to fully contacting the bleeding sites and accessing deep bleeding sites, the hemostat should be capable of rapidly activating hemostatic pathway to efficiently control bleeding [1]. Traditional hemostasis methods involve the addition of allogeneic blood components, which are strongly related to security problems. For instance, the blood components fibrinogen and platelets increase the risk of viral infection when used as hemostats [19]. Previous research has determined that hemostatic pathways are gradually activated by the release of procoagulants, such as microvesicles and adenosine diphosphate (ADP), from erythrocytes (known as red blood cells (RBCs)) to activate platelet aggregation and then thrombosis [20, 21]. Microvesicles initiate coagulation by directly activating factor XII (FXII) or prekallikrein, thereby triggering the coagulation cascade [22], whereas ADP can be liberated following shear stress-related lytic or sublytic injury in RBCs to dramatically affect platelet adhesion and induce platelet aggregation [23]. Therefore, hemostats that can physically puncture RBCs might be able to trigger the rapid release of procoagulants to facilitate blood coagulation [24, 25].

Recently, natural plant pollen has become one of the most widely used encapsulants, making it an ideal hemostatic platform because of various health benefits, such as safety following consumption, reproducibility, physicochemical robustness, and uniform structure [26, 27]. Specifically, meteor hammer-like sunflower pollen with ordered nanospikes has been attractive because it can drill into cells [28]. In a previous study, a magnetic sunflower pollen grain (SPG) was fabricated as a microperforator to puncture the cytomembrane of cancer cells and efficiently deliver payloads, causing the death of cancer cells [14]. Therefore, the inherent geometry of natural sunflower pollen enables it to puncture blood cells for hemostasis in complex wounds with deep and secluded hemorrhagic sites.

**Figure 1:**
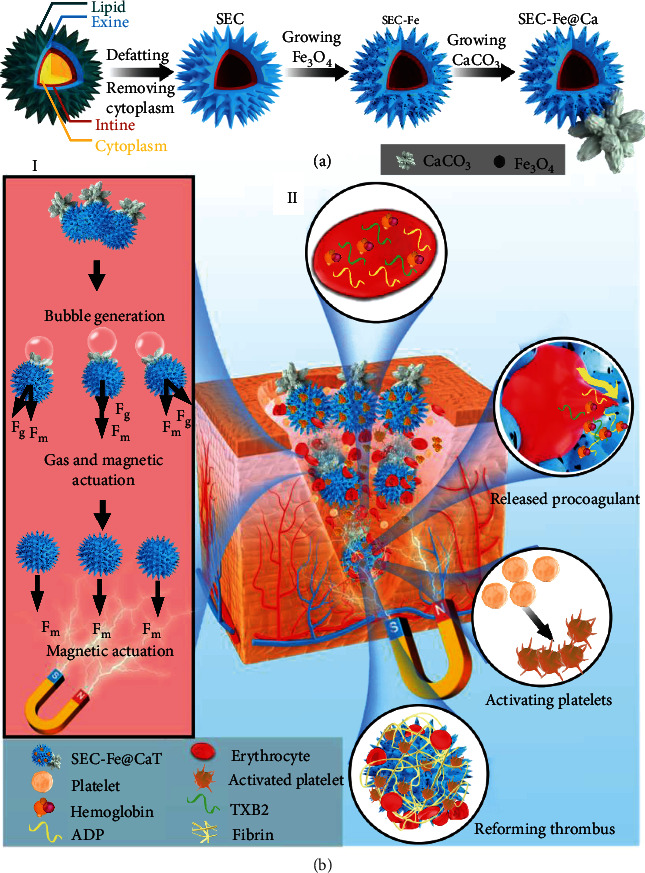
(a) Fabrication procedure of SEC-Fe@Ca. (b) Hemostatic mechanism of SEC-Fe@CaT.

In this study, we developed a meteor hammer-like Janus particle with dual-driven motion (bubble-driven complete self-diffusion in the blood flow and MF-mediated- driven for reverse movement against countercurrent blood flow) using sunflower pollen to puncture RBCs and target deep and secluded hemorrhagic sites. We hypothesized that the treated sunflower pollen could act as meteor hammers to attack and puncture RBCs and thus promote the release of endogenous procoagulants. The meteor hammer-like hemostat was synthesized by treating natural sunflower pollen by acidolysis, followed by *in situ* loading of Fe_3_O_4_; additionally, one side of the pollen matrix was modified with flower-shaped CaCO_3_ clusters. By combining the hemostat with TXA-NH_3_^+^, CaCO_3_ released CO_2_ bubbles to promote the lateral diffusion of the hemostats, whereas the *in situ* loaded Fe_3_O_4_ responded to an applied MF, allowing the hemostat to approach into deep and secluded hemorrhagic points, during which the hemostat collided with RBCs and punctured them easily in the countercurrent blood flow. The punctured RBCs were predicted to trigger the rapid release of procoagulants, thus activating the coagulation cascade and achieving hemostasis (Figure 1). The puncturing performance and propelling properties of the dual-driven hemostats were evaluated, and the mechanism of hemostasis was investigated systematically as well. The intelligent hemostats have immense potential for facilitating hemostasis, as they could fully and extensively contact the irregular bleeding cavity, possess a lasting driving force to access deep bleeding sites, and activate endogenous hemostatic pathways rapidly [29]. This was the first attempt using geometric structure of sunflower pollen to puncture RBCs for hemostasis. The dual-driven mode demonstrated in the present study may offer a total new perspective in drug delivery in blood flow for rapid hemostasis.

## 2. Methods

### 2.1. Materials

Sunflower pollen grains were purchased from Berer Co. Ltd. (Mongolia, China). Phosphoric acid (85% w/v) was supplied by Macklin Biotechnology Co. Ltd. (Shanghai, China). Adenosine diphosphate (ADP) and thromboxane B_2_ (TXB_2_) kits were supplied by Elabscience Biotechnology Co. Ltd. (Wuhan, China). Alexa Fluor® 647 Hamster Anti-Mouse CD61 and Alexa Fluor® 647 Hamster Anti-Mouse CD62P were obtained from Bio-Rad Laboratories Co. Ltd. (Shanghai, China). Unless otherwise indicated, all chemicals were obtained from Sigma-Aldrich Co. Ltd. (Chengdu, China).

### 2.2. Fabrication of Sunflower Sporopollenin Exine Capsules

Hollow sunflower sporopollenin exine capsules (SEC) were prepared in two steps, as previously reported: defatting and cytoplasm removal [27, 30]. Natural sunflower pollen (40 g) was defatted in 200 mL of acetone (50 °C, 8 h) with gentle stirring. Afterwards, to remove internal core substances, the defatted sunflower pollen (approximately 5 g) was suspended in KOH aqueous solution (6%, w/v) while stirring (500 rpm, 85 °C, 6 h). After centrifugation to remove the suspension, the treated sunflower pollen was washed sequentially with acetone, alcohol, and deionized water. Following alkaline lysis, the treated pollen was resuspended in phosphoric acid (100 mL) at 85 °C while stirring (500 rpm) for 6 h. The sample was subjected to acidolysis and centrifugation, and the pollen was collected and washed sequentially, as described above. Finally, hollow sunflower SECs were obtained by freeze-drying.

### 2.3. Preparation of SEC-Fe@CaT

First, SEC loaded with Fe_3_O_4_ (SEC-Fe) was synthesized by *in situ* assembly of Fe_3_O_4_ nanoparticles on the SECs. SECs suspended in deionized water (2 wt%) were prepared. Then, FeCl_2_·6H_2_O and FeCl_3_·4H_2_O (mass ratio of 2 : 1) and 10 mL of NaOH (1 mol/L) were added dropwise at 50 °C for 30 min. The total mass (0.2, 0.6, and 1.8 g) of FeCl_2_·6H_2_O and FeCl_3_·4H_2_O was varied to adjust the final *in situ* loading of Fe_3_O_4_ on SECs. The above suspension was washed in sequence using deionized water, alcohol, and deionized water to remove redundant Fe_3_O_4_. Finally, SEC-Fe was finally obtained after drying under vacuum at 50 °C for 24 h.

Second, a facile precipitation method was used to grow CaCO_3_ over SEC-Fe (SEC-Fe@Ca) [31]. First, the carboxyl group on the SEC was complexed with Ca^2+^ [32]. Second, cetyltrimethylammonium bromide (CTAB) formed rod-like micelles which were linked to the surface of SEC-Fe through electrostatic interaction. Finally, Ca^2+^ reacted with CO_3_^2-^ to form a flower-type CaCO_3_ cluster using CTAB as a template. In this reaction, CaCl_2_, CTAB, and SEC were used as the calcium source, template, and substrate, respectively [33–35]. CTAB/CaCl_2_ solution was prepared by adding CTAB (2 mg/mL) and CaCl_2_ (0.22 M) to 500 mL of a mixture of glycol/water (1 : 1 in volume) and stirring the mixture for 5 min. Afterwards, 4 g of SEC-Fe was added to the above mixture, followed by addition of 500 mL of Na_2_CO_3_ under vigorous stirring for 60 min at 35 °C. The assembled SEC-Fe@Ca in the suspension was collected by magnetic absorption whereas unconjugated CaCO_3_ and Janus particles with weak magnetism were screened out. Finally, the SEC-Fe@Ca Janus particles were washed and freeze-dried.

To prepare SEC-Fe@Ca loaded with thrombin (SEC-Fe@CaT), 0.5 g of SEC-Fe@Ca Janus particles were immersed in thrombin solution, followed by vacuuming three times for 5 min each and lyophilization for 48 h until a stable weight was reached.

### 2.4. Characterizations

The organic element content was determined using elemental analysis (EA) (EA 2400 II, PerkinElmer, USA). Because the nitrogen content of various proteins was 16%, the amount of protein in the samples was obtained by multiplying the percentage of nitrogen element by 6.25 [36]. The morphology, structure, and elemental maps of the samples were determined by scanning electron microscopy (SEM) (Sigma 300, Zeiss, German) with energy dispersive X-ray spectroscopy (EDS). A fluorescence microscope (IX73P2F, Olympus, Japan) was used to collect optical and fluorescence microscopy images. Other characteristics were determined using Fourier transform infrared spectroscopy (FT-IR) (Vertex 70, Bruker, USA), X-ray photoelectron spectroscopy (XPS) (PHI-5000 Versaprobe III, ULVAC-PHI, Japan), X-ray diffraction (XRD) (SmartLab, Rigaku, Japan), and flow cytometry (FCM) (MoFlo Astrios EQ, Beckman Coulter, USA).

### 2.5. Bubble-Driven Dispersibility

The bubble-driven dispersibility of SEC-Fe@Ca was determined in deionized water. SEC-Fe@Ca was initially mixed with TXA-NH_3_^+^ (SEC-Fe@Ca(+t)) in a molar ratio of 1 : 1 (CaCO_3_ to TXA-NH_3_^+^). This mixture (20 mg) was added to water to observe the motion of the SEC-Fe@Ca.

The dispersibility of SEC-Fe@Ca was also assessed by measuring the maximum upstream distance in different directions. A glass tube (inner diameter of 4 mm) filled with stagnant PBS (pH 7.4) was fixed at 90°, 45°, 0°, -45°, and -90°, where 0° is the horizontal direction, and the positive and negative angles represent specific angles above and below the horizontal line, respectively. After the Janus particles with TXA-NH_3_^+^ were fed into the top orifice, the maximum upstream distance was recorded.

### 2.6. MF-Mediated Motion

The MF-mediated motion of SEC-Fe@Ca was investigated using static fluid and flowing liquid models. In the static-fluid model, SEC-Fe@Ca was actuated with an applied MF of 0.4 T in a glass tube filled with PBS (pH 7.4), during which the MF-mediated motion of an individual Janus particle was photographed under an inverted microscope (IX73P2F, Olympus). In the flow-liquid model, the ability of the applied MF to control the movement of SEC-Fe@Ca(+t) (abbreviated as SEC-Fe@Ca(+t+M)) against flowing fluid was investigated. A fluid-propelling device was constructed in-house as we previously reported [13]. The device comprised a glass tube connected to an injection pump at one end and Y-shaped bifurcated junction at the other end. The tube was filled with flowing PBS (pH 7.4), and the fluid propulsion velocity was controlled in the range of 0 to 30 mm·s^−1^ using a pump. After the Janus particles were injected into the Y-shaped tube, the maximum MF-mediated distance in the vertical and horizontal directions was evaluated by changing the distance between the magnet and injection point.

### 2.7. Puncturing Performance

To evaluate the puncturing performance, 10 mg of SEC-Fe@Ca and 10 mg of TXA-NH_3_^+^ in a glass tube filled with RBC solution (RBCs in PBS, 33 vol%) were actuated with an applied MF (B = 0.4 T). After washing the unabsorbed RBCs by washing 3 times, the remaining RBCs were fixed by using glutaraldehyde. SEM (Sigma 300) images of the SEC-Fe@Ca with punctured RBCs were captured.

Studies have shown that injured RBCs can promote the release of ADP and conversion of TXA_2_ to TXB_2_. Therefore, the concentrations of TXB_2_ and ADP in the supernatant were determined after the SEC-Fe@Ca treatments. SEC-Fe@Ca with TXA-NH_3_^+^ (10 mg of SEC-Fe@Ca and 10 mg of TXA-NH_3_^+^) were initially warmed (37 °C, 5 min) and then added to anticoagulant blood (2 mL) and 40 *μ*L of CaCl_2_ solution (0.22 M). After cultivation (37 °C, 10 min) and centrifugation (3000 rpm, 10 min), the supernatant was carefully collected. According to the manufacturer's directions, the concentrations of TXB_2_ and ADP were determined by measuring the absorbance at 525 nm. Natural pollen, SEC, SEC-Fe, and SEC-Fe@Ca without TXA-NH_3_^+^ were also tested using the same procedure.

### 2.8. Platelet Activation

After SEC-Fe@Ca treatment, platelet activation was determined using flow cytometry. SEC-Fe@Ca (5 mg/mL) and TXA (5 mg/mL) were incubated with murine blood for 4 min. Next, AF647-CD61 and AF647-CD62P antibodies were used to stain the mixture in the dark (37 °C, 20 min) to identify mouse platelets and activated platelets, respectively. A flow cytometer (MoFloAstrios EQ, Beckman Coulter, America) was used to measure platelet activation via fluorescence-activated cell sorting. Finally, the expression of CD61 and CD62P was analyzed using FlowJo X software (TreeStar, Ashland, OR, USA). Platelet activation was evaluated using the same procedure for groups treated with SEC-Fe@Ca (5 mg/mL), and CaCl_2_ (0.2 M) as well as untreated groups.

### 2.9. Occlusion Performance in an *In Vitro* Wound-Bleeding Model

*In vitro* hemostasis performance was initially evaluated in whole blood clotting experiments (Supplementary Information, Method 1). The wound-bleeding model was used to evaluate occlusion performance. The device simulating wound was composed of polyacrylamide hydrogel and constructed in-house using a traditional reverse mold technique. The device contained a transparent tube as the simulated vascular vessel and a connected V-shaped gap in the polyacrylamide hydrogel as the simulated bleeding wound. During the test, blood was pumped into the stimulated vascular vessel at a speed of 4 mm/s. After fixing the magnet under the wound-bleeding model, particles were added to the mold through the stimulated V-shaped wound. The time at which bleeding stopped at “the wound” was recorded as the coagulation time.

### 2.10. Blood Coagulation Mechanism

To determine the coagulation mechanism, activated partial thromboplastin time (APTT) and prothrombin time (PT) assays were performed as reported previously [37]. Furthermore, also as previously reported, a whole blood coagulation assay was conducted [37]. In short, particles (10 mg) were mixed with anticoagulant blood and CaCl_2_ (60 *μ*L, 0.2 M), and the blood coagulation time was recorded.

### 2.11. Hemostatic Performance *In Vivo*

In this study, the animal tests conducted were approved by Animal Care and Ethics Committee of Southwest University. Hemostatic performance *in vivo* was evaluated using a rat tail amputation model and a femoral artery hemorrhage model, as previously described [38]. In the tail amputation model, after anesthetizing the rats by pelltobarbitalum natricum solution (50 mg/kg) and cutting the tail vein and femoral artery of the rats, 100 mg of SEC-Fe@Ca powder with 100 mg of TXA-NH_3_^+^ was promptly applied to the wounds. Hemostatic performance was evaluated using the same procedure for groups treated with Celox™ hemostastic powder (100 mg), SEC-Fe@CaT (100 mg), and no treatments. In the femoral artery model, after anesthetizing and cutting the femoral artery of the rats, 100 mg of SEC-Fe@Ca powder with 100 mg of TXA-NH_3_^+^ was applied to the wounds to stop bleeding under an applied MF. Other groups were treated with gauze, Celox™ hemostatic powder, SEC-Fe@CaT, and no treatments. Each group consisted of 6 rats.

To confirm the rapid hemostasis facilitated by SEC-Fe@CaT, a rabbit liver hemorrhage model was used. After anesthetizing the rabbit, the liver was exposed and a V-shaped wound was created. SEC-Fe@CaT (200 mg) with TXA-NH3+ (200 mg) was immediately deposited to stop bleeding under an applied MF, and the blood clotting time was recorded. The wound was treated with gauze, Celox™, and SEC-Fe@CaT using the same procedure. The rabbits were euthanized after the treatment. Then, the livers were fixed in formalin, dehydrated, and sliced, followed by hematoxylin-eosin (H&E) staining following established protocols.

### 2.12. Biocompatibility *In Vivo*

The biocompatibility of SEC-Fe@Ca was evaluated using cytotoxicity (Supplementary Information, Method 2), hemolysis (Supplementary Information, Method 3), and rabbit subcutaneous implantation assays as previously reported [24]. Briefly, 100 mg of the particles were implanted into the back subcutaneous muscle of the rabbits. At 2, 4, 6, 8, 10, and 14 weeks after treatment, the rabbits were euthanized for H&E staining. Celox™ was used as the blank treatment.

### 2.13. Statistical Analysis

Statistical analyses were performed using one-way analysis of variance. Differences were considered statistically significant when *P* value was less than 0.05.

## 3. Results and Discussion

### 3.1. Chemical and Physical Structure of SEC-Fe@ca

**Figure 2:**
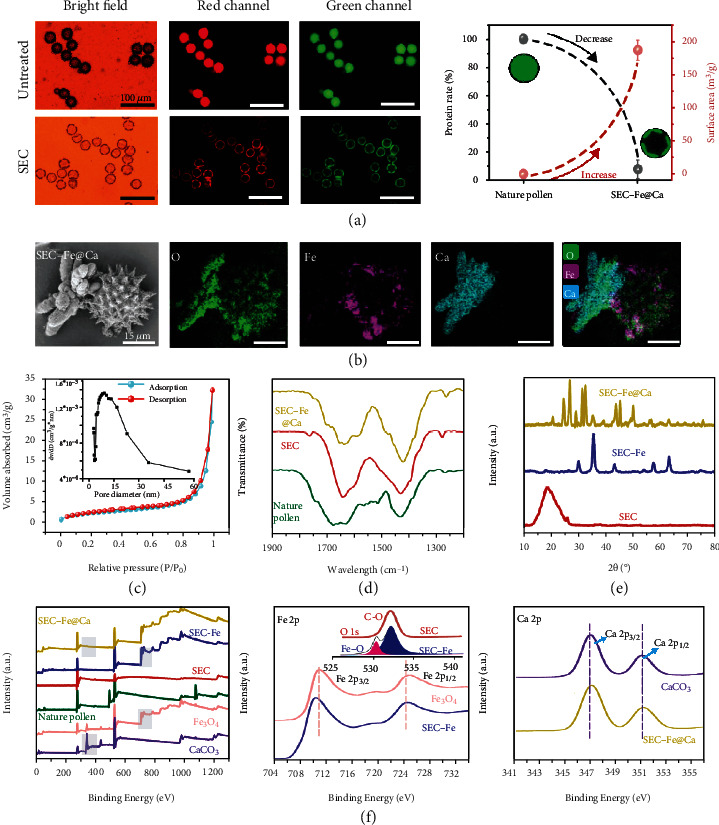
(a) Fluorescence microscope images, protein rate, and specific surface area of natural pollen and SEC. (b) SEM images and EDS mapping of SEC-Fe@Ca. (c) Volume absorption and desorption of SEC-Fe@Ca. Inset: pore diameter analysis. (d) Infrared spectrogram of natural pollen, SEC, and SEC-Fe@Ca. (e) X-ray diffraction (XRD) spectra of SEC, SEC-Fe, and SEC-Fe@Ca. (f) X-ray photoelectron spectroscopy (XPS), Fe 2p, and Ca 2p spectra. Inset: O 1 s spectrum.

As shown in Figure 1, SEC-Fe@Ca was prepared by performing defatting and lysis treatments to effectively remove the cytoplasm and lipid compounds from natural sunflower pollen. The defatting process removes contaminants and attached proteins and lipid compounds, which can cause allergic reactions [39]. After continuous alkaline lysis and acidolysis treatments, the cytoplasmic materials were removed, as confirmed using attenuated fluorescence intensity measurements for SEC, as shown in Figure 2(a). In addition, SEC with removed protein was examined using elemental analysis, revealing that 92% of the protein was removed. Notably, as shown in Figure 2(d), a distinct absorption band at 1690 cm^−1^ allocated to the carboxylic acid group (C=O) was observed in the FT-IR spectrum of SEC (full FT-IR spectrum is shown in Figure S1). Therefore, the carboxyl groups on the surface of SEC were likely exposed after the lipid removal [40]. Moreover, SEC showed visible pores on the surface (Figure 2(a) and SEM image in Figure S2A), significantly increasing the surface area from 0.087 m^3^/g before treatment to 210 m^3^/g after treatment (Figure 2(c)). Previous studies showed that an increased surface area can facilitate the absorption of RBCs and platelets to achieve hemostasis [41]. Therefore, SEC with intact nanospikes, high surface areas, and inner cavities is a promising material for thrombin encapsulation applications.

To endow SECs with magnetic responsiveness, Fe_3_O_4_ was loaded *in situ* to produce SEC-Fe. The X-ray diffraction (XRD) pattern of SEC-Fe (Figure 2(e)) showed characteristic peaks at 30.2°, 35.6°, 43.1°, 53.8°, 57.2°, and 62.7°, corresponding to the (220), (311), (400), (422), (511), and (440) crystallographic nucleation planes of Fe_3_O_4_ [42]. Different concentrations of Fe_3_O_4_ were loaded into SEC to prepare SEC-Fe. The SEM images showed that as the concentration of Fe_3_O_4_ was increased, the structure of SEC-Fe collapsed, particularly at a loading of 43%. Considering the intact nanospike and the hemostatic performance (Figures S2A–S2C), the optimal loading concentration was 24% (w/w) of Fe_3_O_4_, which was used for subsequent investigations.

To achieve widespread contact with bleeding sites, Janus particles (SEC-Fe@Ca) composed of SEC-Fe and self-assembled flower-type CaCO_3_ cluster were successfully fabricated using a facile precipitation method (optical microscope and SEM images in Figure S3), as demonstrated by the EDX images in Figure 2(b). Crystallization of the Janus particles was confirmed using the XRD patterns (Figure 2(e)) in which the characteristic peaks at 21.0°, 25.1°, 27.1°, 32.9°, 50.1°, 55.6°, 72.0°, and 73.6° corresponded to the (004), (110), (112), (114), (118), (224), (1112), and (414) crystal faces of CaCO_3_ vaterite [43]. Moreover, the interactions among the components of the Janus particles, including the SEC, Fe_3_O_4_ nanoparticles, and CaCO_3_ vaterite, were investigated using XPS, as shown in Figure 2(f). In the Fe_3_O_4_ spectrum, two peaks at 724.8 and 711.1 eV were detected, which were assigned to Fe 2p_1/2_ and Fe 2p_3/2_, respectively [44]. When SEC was loaded *in situ* with Fe_3_O_4_, the peaks for Fe 2p_1/2_ and Fe 2p_3/2_ in the SEC-Fe spectrum shifted to lower binding energies of 724.1 at and 710.1 eV, respectively. In contrast, the characteristic O 1 s peak dramatically increased from 513.8 eV in the SEC spectrum to 532.3 eV (fitted peak assigned to C-O) in the SEC-Fe spectrum. Notably, the characteristic peak of Fe–O–Fe was detected after peak fitting analysis of the SEC-Fe spectrum. The change in the binding energies of the O 1 s and Fe 2p peaks likely originated from complexation of the carboxyl group with metal ions, which changed their chemical environments. Oxygen atoms tend to lose electrons and share their lone pair electrons with Fe atoms in the complexation process, thus decreasing the electron cloud density of O 1 s and increasing the binding energy of O 1 s while increasing the electron cloud density of Fe 2p and decreasing the binding energy of Fe 2p [45].

### 3.2. Bubble-Driven and MF-Mediated Motion

**Figure 3:**
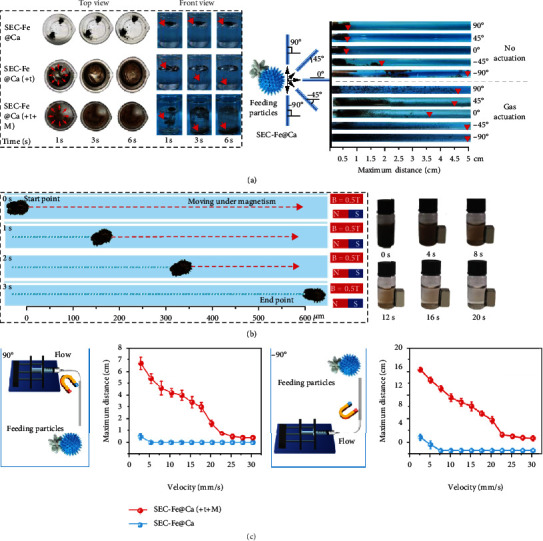
(a) Motion of SEC-Fe@Ca under different conditions and schematic of SEC-Fe@Ca particles in tubes fixed at different angles to observe the travel distance. (b) Microscope images of magnetic field-mediated motion of SEC-Fe@Ca in static water and collection of SEC-Fe@Ca under an external magnetic field. (c) Maximum upstream distance of SEC-Fe@Ca and SEC-Fe@Ca(+t+M) in water flowing at different flow rates at 90° and -90° feeding angles.

CaCO_3_ was incorporated into the Janus particles to endow the particles with bubble-driven capacities because CO_2_ bubbles were generated in the presence of TXA-NH_3_^+^. The images from the top and front views (Figure 3(a)) show that SEC-Fe@Ca reacted rapidly with TXA-NH_3_^+^ to generate CO_2_ bubbles upon contact with water. When the bubbles were released in one direction, the generated propulsion force pushes the particles in the opposite direction. Because the bubbles were released in random directions for each particle, the motion of the particles vary to achieve a full distribution in 6 s (Video S1). To further clarify the behavior of Janus SEC-Fe@Ca particles in an acidic solution, optical microscopy was performed to observe the evolution of bubbles and motion of the particles. The generation, growth, separation of bubbles and self-propelling behavior of the particles is shown in Figure S4 and Video S2. In addition, the maximum distance travelled by the particles in different directions was investigated using an in-house model. The results convincingly showed that the Janus particle with bubble propulsion travelled an appreciably longer distance (more than 3.5 cm) than that without bubble propulsion (approximately 1 cm) at angles 0°, 45°, and 90°. Although there was no significant movement in the -90° direction because of gravity, the Janus particles with bubble propulsion showed a remarkably homogeneous dispersion.

MF-mediated motion allows SEC-Fe@Ca to travel sustainably against the bloodstream and access deep bleeding sites. The MF-mediated motion of SEC-Fe@Ca was monitored by microscopy, showing that the particles achieved approximately 200 *μ*m/s under a 0.5 T magnetic field (Figure 3(b), Video S3). These results indicate that the movement of SEC-Fe@Ca can be easily controlled using an applied MF. Moreover, to verify the dual-driven motion of SEC-Fe@Ca against flow, the maximum upstream distances were recorded against different flow velocities of 0 to 30 mm/s. As shown in Figure 3(c), when the flow was perpendicular to the horizontal direction (-90°), the maximum upstream distance for SEC-Fe@Ca(+t+M) with dual-driven motion reached 16 cm against a flow velocity of 2.5 mm/s and then linearly decreased as the flow velocity increased. In contrast, because of gravity, SEC-Fe@Ca, without a driving force, traveled marginally downward. When the flow velocity was 7.5 mm/s and higher, the particles were unable to move against water flow and were washed away immediately by the current. Similar trends were observed in the 90° flow direction, indicating superior upstream propulsion for SEC-Fe@Ca with dual-driven motion. In addition, to avoid the influence of gravity, the maximum upstream distances of SEC-Fe@Ca and SEC-Fe@Ca(+t+M) were evaluated at an angle of 0°. Similarly, SEC-Fe@Ca with dual-driven motion exhibited superior upstream propulsion as shown in Figure S5.

### 3.3. Puncture of RBC and Platelet Activation

**Figure 4:**
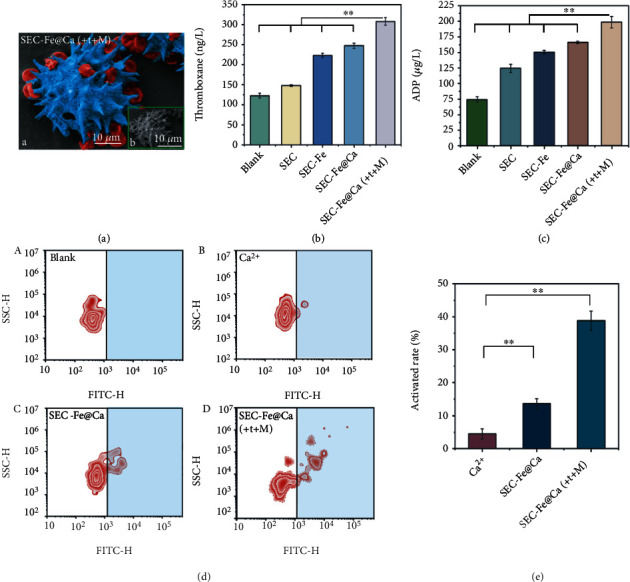
(a) (A) Pseudocolor SEM images and (B) SEM image of RBCs treated with SEC-Fe@Ca(+t+M). Red pseudocolor represents RBCs and blue pseudocolor represents SEC-Fe@CaT. (b) Release of thromboxane B_2_ and (c) adenosine diphosphate. (d) Platelet activation rate treated with (A) blank, (B) CaCl_2_, (C) SEC-Fe@Ca, and (D) SEC-Fe@Ca(+t+M). (e) Rate of activated platelets.

RBCs play an integral role in blood clotting; however, their functions in thrombus formation and hemostasis are not well-understood [20, 25, 46]. RBCs contribute to the formation of thrombi and participate in RBC aggregation, coagulation activation, fibrinogen combination, and thrombin generation. More importantly, ADP and other factors released from hemolytic or damaged RBCs can activate platelet aggregation and then thrombosis [20, 21]. Thus, the capacity of the particles to puncture RBCs was examined (Figure 4(a)). RBCs were either punctured by spikes or attached to the surface upon exposure to SEC-Fe@Ca with bubble propulsion. Simultaneously, echinocytes were also detected, as shown in Figure S6. This result supports the presence of ADP, transformed from adenosine triphosphate (ATP), as echinocytes are produced by RBCs while consuming large amounts of ATP [47]. ADP content was determined using an enzyme-linked immunosorbent assay, and the results are presented in Figure 4(c). The concentrations of ADP in whole blood were as high as 198 *μ*g/L after exposure to bubble and MF-driven SEC-Fe@Ca, which was nearly three-fold that in unexposed blood (without treatments). This discrepancy in the ADP concentration may have originated from the larger number of procoagulants released from the damaged or punctured RBCs. The TXB_2_ content showed a similar trend as the ADP content, as the TXB_2_ content in whole blood exposed to dual-driven SEC-Fe@Ca (308 ng/L) was greater than that in unexposed blood (blank group, 132 ng/L) and blood exposed to SEC-Fe@Ca with no propulsion (Figure 4(b)). TXB_2_, released by activated platelets, stimulates vasoconstriction and reduces blood flow at the site, leading to clot formation. Thus, dual-driven SEC-Fe@Ca can facilitate the release of ADP and TXB_2_ to promote the formation of clots and stop bleeding.

According to previous reports, hemolytic and damaged RBCs release procoagulants, such as hemoglobin and ADP, which can induce the activation of platelets to facilitate the formation of clots. Thus, platelet activation was investigated using flow cytometry, as shown in Figures 4(d) and 4(e). Platelet activation after exposure to Janus particle SEC-Fe@Ca with dual-driven motion reached as high as 39.4%, which was dramatically higher than that after exposure to the Janus particle without propulsion (13.6%) and almost eight-fold greater than that after exposure to Ca^2+^ (4.8%). One possible reason for the high rate of platelet activation by dual-driven SEC-Fe@Ca is that sharp thorns on SEC-Fe@Ca can puncture RBCs and the other possible reason is that TXA-NH_3_^+^ and the MF may provide the driving force to increase the possibility of the particle colliding with the RBCs. Both of these factors facilitate the puncture of RBCs by particles, causing them to release procoagulants that enhance platelet activation. Moreover, the high platelet activation rate may be attributed to the high specific surface area of SEC-Fe@Ca, which ensures rapid absorbance of exudates and prevents the escape of RBCs and platelets. The geometric structure also promotes the adhesion of platelets, RBCs, blood proteins, and other active procoagulants to induce fibrin clot formation and accelerate blood coagulation.

These results suggest that because of their propulsion properties and geometric structure, meteor hammer-like Janus particles can aggregate and puncture RBCs to release procoagulants and can adhere to coagulation materials to activate platelets. These properties collectively contribute to their superior hemostatic capacity.

### 3.4. Hemostasis Evaluation *In Vitro*

**Figure 5:**
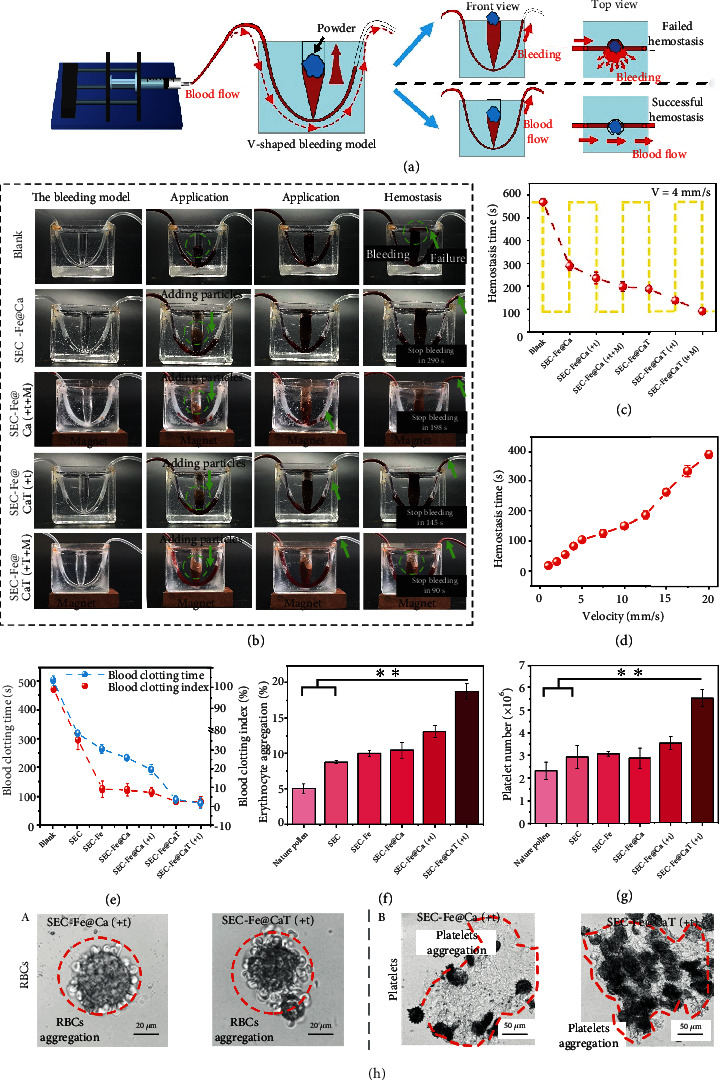
*In vitro* hemostasis evaluation model. (a) Schematic of the in vitro bleeding model. (b) Photographs of hemostatic processes of different particles in the V-shaped bleeding model at a velocity of 4 mm/s. (c) Hemostasis time of different particles at a velocity of 4 mm/s. (d) Average hemostasis time with SEC-Fe@CaT(+t+M) in V-shaped bleeding model at different velocities. (e) Blood clotting index and blood clotting time with different particles. (f) The amount of RBCs adhered. (g) Number of platelets adhered. (h) Microscopic images of (a) adhered RBCs (enclosed by red dashed line) and (b) adhered platelets (enclosed by red dashed line) after exposure to SEC-Fe@Ca(+t) and SEC-Fe@CaT(+t).

Wounds with bleeding at deep and imperceptible sites are often hard to treat using conventional ways. To investigate the hemostatic capacity of SEC-Fe@CaT in complex hemorrhage wounds, a V-shaped wound (Figure 5(a)) was designed to simulate real bleeding *in vitro* and *in vivo*. The image in Figure 5(a) shows the microinjector used to introduce and control the flow velocity of injected fresh blood in the hemostatic model to investigate the hemostatic performance of particles at different flow velocities. The following five groups were investigated: (A) Blank, (B) SEC-Fe@Ca, (C) SEC-Fe@Ca(+t+M), (D) SEC-Fe@CaT(+t), and (E) SEC-Fe@CaT(+t+M). Hemostasis was considered successful when the blood flowed into the right infusion tube. In contrast, when blood leaked from the V-shaped wound rapidly, hemostasis was considered unsuccessful.

As shown in Figures 5(b) and 5(c), when the velocity of blood flow was set to 4 mm/s, SEC-Fe@Ca achieved hemostasis in 290 s, which was significantly faster than that achieved by the blank group in which natural coagulation occurred after 600 s to stop bleeding. Thus, the spike structure for puncturing RBCs and large specific surface area for adhering RBCs, platelets, and coagulation materials collectively contributed to hemostasis. In addition, the hemostatic performance of SEC-Fe@Ca was significantly enhanced when dual drive was applied. The hemostasis time with SEC-Fe@Ca(+t+M) (198 s) was almost similar to that with SEC-Fe@CaT (187 s). Thus, propulsion promoted the formation of clots in deep hemorrhagic sites. As reported previously, because SEC has numerous nanopores, a large specific surface area, and an internal cavity, it can be applied in drug delivery [36]. Thus, to achieve faster hemostasis, SEC-Fe@Ca with an optimal thrombin loading of 13.6 U/g, abbreviated as SEC-Fe@CaT, was prepared (thrombin-loading efficiency is shown in Figure S7). SEC-Fe@CaT with dual-driven motion, abbreviated as SEC-Fe@CaT(+t+M), facilitated the formation of a thrombus to block the hemorrhagic spot, thus achieving fast hemostasis in 90 s.

Similarly, Figure 5(d) shows the outstanding hemostatic performance of SEC-Fe@CaT(+t+M) at different blood flow velocities. Hemostasis was achieved in a short time (less than 200 s) at a low blood flow velocity (less than 10 mm/s). Although hemostasis was achieved for a longer time as the blood flow rate increased, even at a blood flow of 20 mm/s, SEC-Fe@CaT(+t+M) blocked the wound to achieve hemostasis within 400 s. In addition, excellent *in vitro* hemostatic performance was demonstrated. As shown in Figures 5(e)to 5(g), SEC-Fe@CaT(+t) led to the shortest whole blood coagulation time (72 s), lowest blood clotting index (2%), and the largest numbers of adhered RBCs (18.7%) and platelets (5.2 × 10^6^) among the hemostats investigated. These results support that thrombin loading, nanospikes for the release of procoagulants to promote the formation of thrombi, and a large specific surface area for adhering blood cells collectively contributed to rapid hemostasis. Similar results (Figure 5(h) and Figure S8) were obtained for SEC-Fe@CaT(+t), in which optical and SEM images revealed their capacity to adhere to a large number of RBCs and platelets.

According to the results described above, the excellent hemostatic performance of SEC-Fe@CaT(+t+M) originates from its capacity to move under the action of an applied MF and gas propulsion, i.e., the particles travelled deep into the wound and simultaneously promote the uniform dispersal of thrombin [12, 13]. Additionally, the release of procoagulants and thrombin contributed to platelet activation and the accelerated formation of blood clots.

**Figure 6:**
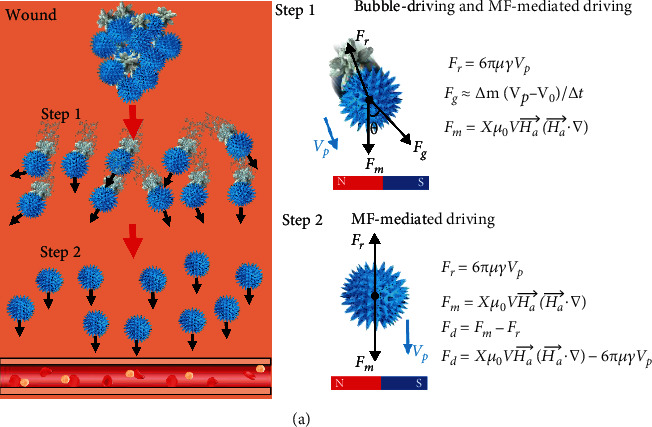
(a) Schematic of the motion of SEC-Fe@Ca(+t+M).

Throughout the process, the particles are subjected to gas propulsion (*F*_*g*_), magnetism (*F*_*m*_), and resistance (*F*_*r*_). The resistance (*F*_*r*_) of the particles is approximated as follows [48]:

(1) $$\begin{array}{c} F_{r}=6\pi \mu \gamma V_{P}, \end{array}$$

where *μ*, *V*_*P*_, and *γ* are the radius of the bubble colloid, velocity of particles, and viscosity of water, respectively. As shown in Figure 6(a), the movement process is divided into two steps. First, SEC-Fe@Ca particles were driven by CO_2_ and the applied MF. When a mixture of TXA-NH_3_^+^ and SEC-Fe@Ca contacted water, coordinated TXA-NH_3_^+^ dissolved quickly to produce an acidic environment, leading to a neutralization reaction between CaCO_3_ and TXA-NH_3_^+^ to generate CO_2_ bubbles on one side of the particles and provide the driving force (*F*_*g*_). According to previous studies, the Reynolds number ranged from 1 to 100 in this system, and the gas propulsion (*F*_*g*_) was approximated according to the following [49]:

(2) $$\begin{array}{c} F_{g}\approx \frac{∆\text{m}\left(V_{P}-V_{0}\right)}{∆t}, \end{array}$$

where Δ*m*, *V*_0_, and Δ*t* are the mass change of CaCO_3_, velocity of CO_2_ bubbles, and the average bubble growth time, respectively. Under the action of an applied MF, with Fe_3_O_4_ on the surface, the particles were subjected to a downward force (*F*_*m*_). Thus, the magnetic force (*F*_*m*_) on the particle increased according to the following [50]:

(3) $$\begin{array}{c} F_{m}=X\mu_{0}V\overset{⟶}{H_{a}}\left(\overset{⟶}{H_{a}}∙\nabla \right), \end{array}$$

where *X*, *μ*_0_, *V*, and *H*_*a*_ are the magnetic susceptibility, the magnetic permeability of free space, total volume of Fe_3_O_4_ on SEC-Fe@Ca, and the applied MF, respectively.

As shown in Step 1 of Figure 6(a), the particles were subjected to bubble repulsion *F*_*g*_ and MF-driven repulsion *F*_*m*_. Because the CO_2_ gas produced at the tail of the particles provides *F*_*g*_ and the direction of *F*_*g*_ is uncertain and random depending on the direction of the bubble, the particles can disperse rapidly by gas propulsion. Different angles (*θ* = 0° to 360°) between *F*_*g*_ and *F*_*m*_ result in different directions for the motion of the hemostats, and thus the hemostats move around and disperse rapidly. This prediction is consistent with the results presented in Figure 3(a).

In Step 2, the particles were subjected to the MF and resistant force *F*_*r*_ when CaCO_3_ was used (Figure 6(a)). When *F*_*m*_ is vertically downward, *F*_*m*_ provides the propulsion against *F*_*r*_, assuming that *μ*_0_, *X*, *V* and *H*_*a*_ are constant. As the distance between the magnet and the particles decreased, the local magnetic field strength ($\overset{⟶}{H_{a}}∙\nabla$) increases, which increases *F*_*m*_, and thus the hemostats can move upstream to deep bleeding sites.

(4) $$\begin{array}{c} F_{d}=F_{m}-F_{r}. \end{array}$$

With dual-drive functionality, the particles disperse horizontally under the repulsion of *F*_*g*_ and move downward to deeper sites under the repulsion of *F*_*m*_.

### 3.5. Hemostasis *In Vivo*

**Figure 7:**
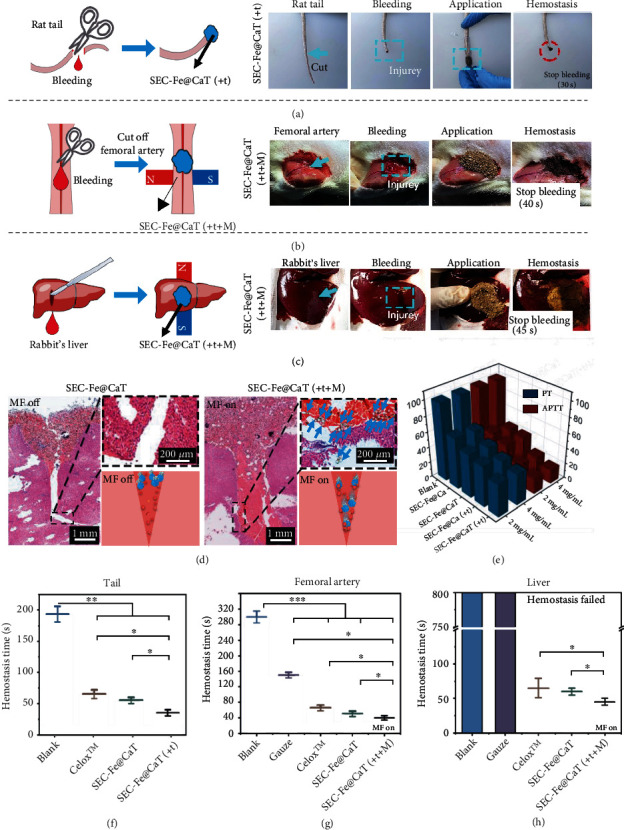
(a) Schematic of rat docking tail model and images of hemostasis process for SEC-Fe@CaT(+t) on rat tail. (b) Schematic of the femoral artery model and images of hemostasis process for SEC-Fe@CaT(+t+M) on femoral artery. (c) Schematic of liver model with a V-shaped wound and *in vivo* hemorrhage process on liver. (d) Histological sections of V-shaped wounds treated with SEC-Fe@CaT and SEC-Fe@CaT(+t+M). The samples are outlined with dotted lines. (e) Prothrombin time (PT) and activated partial thromboplastin time (APTT). (f) Hemostasis time of different particles on rat docking tail, (g) femoral artery, and (h) liver.

Because of the outstanding performance of SEC-Fe@CaT in the *in vitro* hemostatic model, its blood coagulation performance *in vivo* was investigated. As shown in Figure 7, SEC-Fe@CaT showed excellent hemostatic performance. As shown in Figures 7(a) and 7(f), after the application of SEC-Fe@CaT with TXA-NH_3_^+^, abbreviated as SEC-Fe@CaT(+t), the injured tail stopped bleeding in 30 s, dramatically faster than that without any treatments (180 s) and even faster than that following application of Celox™ (67 s). With Celox™, bleeding continued for 30 s (Figure S9).

In addition, to better illustrate the hemostatic performance of SEC-Fe@CaT against severe bleeding from irregular and deep wounds, a V-shaped wound with a deep bleeding point was created in the rat femoral artery and rabbit liver. As shown in Figures 7(b) and 7(g), compared with the other groups, SEC-Fe@CaT(+t+M) significantly enhanced hemostasis. Wound treated with Celox™ and gauze stopped bleeding within 70 and 150 s, respectively. In comparison, SEC-Fe@CaT achieved faster hemostasis (50 s), and SEC-Fe@CaT with dual-driven motion arrested bleeding in 30 s (Video S4). The weak hemostatic properties of gauze and Celox™ likely originate from the difficulty in transporting the hemostat to the bleeding sites. Similarly, in a previous study, using a gelatin sponge as a hemostat did not stop bleeding in complex and deep wounds [51]. As shown in Figures 7(c) and 7(h), SEC-Fe@CaT(+t+M) exhibited the best hemostatic performance with a short hemostasis time of 45 s in the rabbit liver model (Video S5). Images of Celox™ in femoral artery and liver wounds are shown in Figures S10 and S11, respectively.

To determine whether the particles can be propelled into the deep bleeding sites of wounds, the distribution of the hemostat in the V-shaped wound was analyzed using hematoxylin and eosin staining. As shown in Figure 7(d), without propulsion, most SEC-Fe@CaT particles remained on the surface of the wound because of their inability to overcome high-speed blood flow, and thus they failed to treat the bleeding site. In comparison, a large number of SEC-Fe@Ca particles with dual-driven motion were dispersed into the deep area of the V-shaped wound (the particles are marked by the blue arrow). Thus, an abundance of SEC-Fe@CaT particles, driven by an applied MF and CO_2_ gas, can overcome the blood flow and arrive at the deep bleeding sites of complex and deep wounds to significantly accelerate hemostasis.

Based on these results, we predicted that rapid hemostasis with SEC-Fe@CaT was achieved through a multimodal mechanism. First, when SEC-Fe@CaT(+t+M) was applied to the bleeding wound, the production of CO_2_ gas causes the particles to disperse widely, facilitating their contact with the blood site. Second, particles with dual-driven motion easily puncture RBCs to release more procoagulants, promoting platelet activation. Third, SEC-Fe@CaT (with thrombin) can serve as a matrix for adhesion of clotting factors. Finally, particles with punctured RBCs and activated platelets can target deep bleeding sites driven by an applied MF and gas propulsion. They accelerate the formation of strong fiber-reinforced occlusive thrombi in the wound to achieve rapid hemostasis.

PT is a common indicator used to evaluate the functions of hemostat on endogenous pathway of blood coagulation, whereas APTT is used to evaluate the exogenous pathway of blood coagulation [37]. As shown in Figure 7(e), the PT and APTT values of the experimental groups were markedly lower than those of the blank. Furthermore, application of SEC-Fe@CaT(+t) particles resulted in the lowest PT and APTT. These results suggest that SEC-Fe@CaT(+t) particles can promote rapid hemostasis via both endogenous and exogenous coagulation pathways during clotting.

### 3.6. Biocompatibility

Cell activity assays revealed that SEC-Fe@Ca was noncytotoxic. As shown in Figures S12 and 8A, cell activity was greater than 95% after the cells were cultivated with different concentrations of SEC-Fe@Ca for 1, 2, and 3 days. This result indicated that the cell toxicity of SEC-Fe@Ca was grade 0 (relative growth rate ≥ 95%) according to the toxicity rating standard of ISO10993-1 [52]. Thus, SEC-Fe@Ca was considered as noncytotoxic.

**Figure 8:**
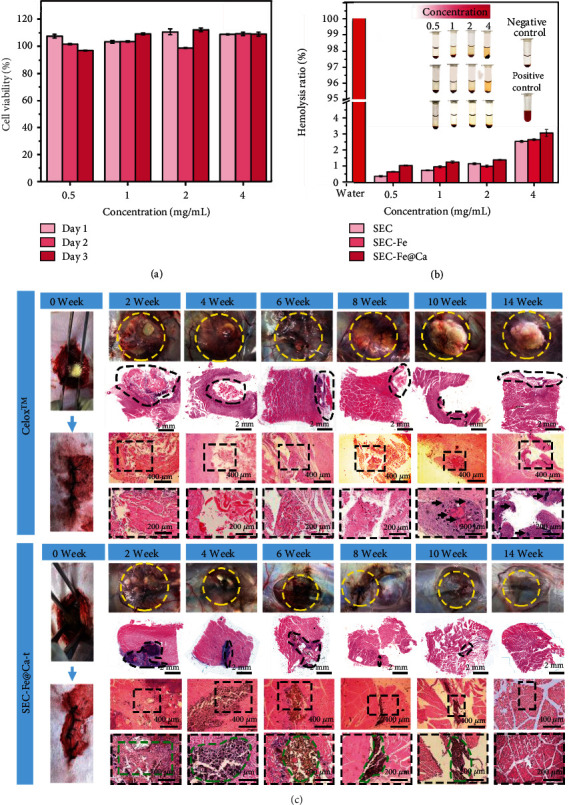
Hemolysis test, *in vivo* cytotoxicity assays, and subcutaneous implantation in the back-muscle assay. (a) Cell viability. (b) Hemolysis ratio of different particles. Inset: images from hemolysis assay. (c) Photographs of the muscular tissue and histological sections after implanting Celox™ and SEC-Fe@Ca(+t).

A hemolysis test was performed to assess the blood compatibility of the hemostats [53]. Images of the different samples in the hemolysis test are shown in the inset of Figure 8(b). Obviously, the solution was shiny red in the positive control group compared to in the other groups, indicating that the RBCs were completely hemolytic. At low sample concentrations, the solutions were transparent, and at high sample concentrations, the solutions were primrose yellow because of slight plasmorrhexis. Thus, SEC, SEC-Fe, and SEC-Fe@Ca induced less plasmorrhexis compared to the positive controls. Figure 8(b) shows the same results as the histogram. Hemostats with a hemolysis ratio of less than 5% are considered blood compatible according to international standards [54]. The hemolysis ratio of all groups was less than 5%, although the concentration of the samples reached up to 10 mg/mL. Hence, the samples were regarded as biosafe materials for RBCs *in vivo*.

Biomaterials used in the human body should be biodegradable to prevent inflammation and other complications [53]. Biocompatibility was tested *in vivo* by subcutaneous implantation of SEC-Fe@Ca (*n* = 6 per group) into the back muscle. Images of the histological sections revealed degradation of SEC-Fe@Ca and Celox™ at 2, 4, 6, 8, 10, and 14 weeks post-surgery (Figure 8(c)). SEC-Fe@Ca(+t) led to slight inflammation (yellow circle) with minor festering in the first week, and festering (yellow circle) disappeared by 4 weeks after surgery. In contrast, Celox™ caused severe inflammation with pustules (yellow circle) and wound festering, with the pustule persisting for 14 weeks. Moreover, the implanted SEC-Fe@Ca degraded gradually over 14 weeks, and SEC-Fe@Ca was not detected in the tissue section after 14 weeks. This result indicated that the material was completely degraded. In contrast, Celox™ particles (black arrow) were detected at 14 weeks. This may be because of the efficient phagocytosis of macrophage cells. The chemical moieties of SEC such as its aliphatic and oxygenated aromatics are likely recognized by pattern-recognition receptors (carbohydrate-binding receptors). This recognition may have induced the activation of phagocytosis of macrophage cells and promoted the phagocytosis of SEC-Fe@Ca by macrophages. Briefly, the results indicate that degradation of SEC-Fe@Ca was relatively rapid compared to that of Celox™.

In addition, the biodistribution and histological changes of SEC-Fe@Ca in rabbits were assessed after a hemostatic experiment *in vivo* (Supplementary Information, Method 4). Different organs from the rabbits 1, 7, and 14 days after hemostasis and tissue histology were evaluated. The images of various organs and tissue section morphologies in Figures S13 and S14 indicated no obvious pathologies, no histomorphological changes, and no inflammatory cell infiltration. As shown in Figure S14B, the concentration of iron ions did not significantly improved in the main organs compared to in those from the blank group (there are significant differences among the four groups (*p* < 0.05)), indicating that SEC-Fe@Ca did not undergo metabolism or continuously accumulated in the organs. In summary, the application of the SEC-Fe@Ca did not lead to systemic toxicity.

The biocompatibility results support the biodegradability and safety of SEC-Fe@Ca, indicating that they can be used as an optimal hemostat for treating complex and deep wounds.

## 4. Conclusion

Rapid hemostasis of deep and impressable wounds remains challenging. Inspired by the attack mode of the meteor hammer, sunflower pollen-based Janus particle SEC-Fe@CaT with bubble and MF-driven motion was designed to treat deep wounds. The Janus particles diffuse and overcome blood resistance to deliver thrombin to deep bleeding sites, while also promoting hemostasis by puncturing RBCs with their spikes to release procoagulants, thus activating platelets. Moreover, Janus particles induced excellent hemostasis in deep and complex wounds *in vivo*. The results show that SEC-Fe@CaT stopped bleeding within 30 and 45 s, respectively, in the femoral artery and liver models. These values were much smaller than those obtained with gauze (150 and 800 s) and commercial Celox™ (70 and 67 s). Histological analysis further demonstrated that SEC-Fe@CaT can move into deep bleeding sites. Furthermore, a subcutaneous implantation assay showed that SEC-Fe@CaT exhibited good biodegradability and biocompatibility *in vivo*. Self-propulsion and magnetic actuation were successfully introduced into the SEC. The geometric structure of the sunflower pollen exhibited dual-driven movement, which facilitated the countercurrent delivery of payloads and released procoagulants from RBCs.

## Data Availability

All data needed to evaluate the conclusions is present in the paper and the supplementary materials. Additional data related to the paper may be requested from the authors.

## Supplementary Materials

Supplementary Materials Figure S1: full Fourier transform-infrared spectra of nature pollen, SEC, and SEC-Fe@Ca. Figure S2: (a) scanning electron microscopy (SEM) images of natural sunflower pollen, SEC, and SEC-Fe with different Fe3O4 loadings. (b) Mass fraction of iron in different samples. (c) In vivo blood clotting time of different samples. Figure S3: optical microscope (a) and scanning electron microscopy (b) images of Janus SEC-Fe@Ca. Figure S4: motion behavior of self-propelling SEC-Fe@Ca in acid situation. Figure S5: maximum upstream distance of SEC-Fe@Ca and SEC-Fe@Ca(+t+M) in water flowing at different flow rates at 0° feeding angles. Figure S6: scanning electron microscopy image of echinocytes treated with SEC-Fe@Ca(+t+M). Figure S7: thrombin activity as a function of loaded thrombin. Figure S8: pseudocolor scanning electron microscopy images of (a) red blood cells and (b) platelets treated with SEC-Fe@CaT(+t) and scanning electron microscopy images. Red pseudocolor represents erythrocytes, brown pseudocolor represents platelets, and blue pseudocolor represents SEC-Fe@CaCO3 particle into SEC-Fe@Ca. Figure S9: hemorrhage control processes of CeloxTM on rat tail. Figure S10: in vivo hemorrhage control processes of CeloxTM on femoral artery. Figure S11: in vivo hemorrhage control processes of CeloxTM on liver. Figure S12: fluorescence and bright field microscopy images of L929 cell cultivated with SEC-Fe@Ca for 1, 2, and 3 days. Figure S13: Various organs from New Zealand white rabbit treated with SEC-Fe@CaT. Figure S14: (a) Tissue section from main organs of rabbit after hemostatic treatment with SEC-Fe@CaT. (b) Concentration of iron ion in the main organs after hemostatic treatment of SEC-Fe@CaT for 1, 7, and 14 days. Video S1: vacroscopic motion of SEC-Fe@Ca in acid solution (pH = 2). Video S2 Bubble-generation and self-propelling behavior of the SEC-Fe@Ca (pHsolution = 2). Video S3 MF-mediated motion of SEC-Fe@Ca. Video S4: evaluation of hemostatic behavior of SEC-Fe@CaT(+t+M) by a rat model of femoral artery hemorrhage. Video S5: evaluation of hemostatic behavior of SEC-Fe@CaT(+t+M) by a rabbit of liver hemorrhage.
