# Supplementary material for: Dual-Driven Hemostats Featured with Puncturing Erythrocytes for Severe Bleeding in Complex Wounds
Source: Research (Wash D C). 2022 May 31;2022:9762746. doi: 10.34133/2022/9762746 (PMC9178490; doi:10.34133/2022/9762746)
Supplement: Supplementary Materials — Figure S1: full Fourier transform-infrared spectra of nature pollen, SEC, and SEC-Fe@Ca. Figure S2: (a) scanning electron microscopy (SEM) images of natural sunflower pollen, SEC, and SEC-Fe with different Fe3O4 loadings. (b) Mass fraction of iron in different samples. (c) In vivo blood clotting time of different samples. Figure S3: optical microscope (a) and scanning electron microscopy (b) images of Janus SEC-Fe@Ca. Figure S4: motion behavior of self-propelling SEC-Fe@Ca in acid situation. Figure S5: maximum upstream distance of SEC-Fe@Ca and SEC-Fe@Ca(+t+M) in water flowing at different flow rates at 0° feeding angles. Figure S6: scanning electron microscopy image of echinocytes treated with SEC-Fe@Ca(+t+M). Figure S7: thrombin activity as a function of loaded thrombin. Figure S8: pseudocolor scanning electron microscopy images of (a) red blood cells and (b) platelets treated with SEC-Fe@CaT(+t) and scanning electron microscopy images. Red pseudocolor represents erythrocytes, brown pseudocolor represents platelets, and blue pseudocolor represents SEC-Fe@CaCO3 particle into SEC-Fe@Ca. Figure S9: hemorrhage control processes of CeloxTM on rat tail. Figure S10: in vivo hemorrhage control processes of CeloxTM on femoral artery. Figure S11: in vivo hemorrhage control processes of CeloxTM on liver. Figure S12: fluorescence and bright field microscopy images of L929 cell cultivated with SEC-Fe@Ca for 1, 2, and 3 days. Figure S13: Various organs from New Zealand white rabbit treated with SEC-Fe@CaT. Figure S14: (a) Tissue section from main organs of rabbit after hemostatic treatment with SEC-Fe@CaT. (b) Concentration of iron ion in the main organs after hemostatic treatment of SEC-Fe@CaT for 1, 7, and 14 days. Video S1: vacroscopic motion of SEC-Fe@Ca in acid solution (pH = 2). Video S2 Bubble-generation and self-propelling behavior of the SEC-Fe@Ca (pHsolution = 2). Video S3 MF-mediated motion of SEC-Fe@Ca. Video S4: evaluation of hemostatic behavior of SEC-Fe@CaT(+t+M) [file 9762746.f1.zip › Supplementary_Information_Haoyu_Qiu.docx]

**Dual-driven hemostats featured with puncturing erythrocytes for severe bleeding in complex wounds**

*Haoyu Qiu ^a,1^, Guangqian Lan ^a,b,1^, Weiwei Ding ^c^, Xinyu Wang ^c^, Wenyi Wang ^d^, Dahua Shou ^d^，Fei Lu ^a,b^, Enling Hu ^a,b,d^, Kun Yu ^a,b^, Songmin Shang ^d,^*, Ruiqi Xie ^a,b,d,^**

^a^State Key Laboratory of Silkworm Genome Biology, College of Sericulture, Textile and Biomass Sciences, Southwest University, Chongqing 400715, China

^b^Chongqing Engineering Research Center of Biomaterial Fiber and Modern Textile, Chongqing 400715, China

^c^Division of Trauma and Surgical Intensive Care Unit, Research Institute of General Surgery, Jinling Hospital, Medical School of Nanjing University, Nanjing 210002, Jiangsu Province, PR China

^d^Institute of Textiles and Clothing, The Hong Kong Polytechnic University, Kowloon, Hong Kong

*Corresponding author at: State Key Laboratory of Silkworm Genome Biology, College of Sericulture, Textile and Biomass Sciences, Southwest University, Chongqing 400715, China

E-mail addresses: xie.ruiqi@connect.polyu.hk

^1^Equally contributed

Supplementary information, method 1

Whole blood clotting experiment in vitro

Fresh blood was collected from healthy New Zealand rabbits and mixed with sodium citrate (3.8% (w/v) sodium citrate solution:blood = 1:9 (v/v)). The prepared samples (50 mg) were placed in a centrifuge tube (5 mL), and 2 mL of fresh anticoagulated whole blood and CaCl_2_ solution (0.2 M) were added to the tube. The blank group contained an additional tube without particles. Subsequently, the centrifuge tubes was incubated at 37 °C while monitoring and evaluating the sample every 15 s. The blood was stagnant when the centrifuge tube was inclined at 45°, and the blood clotting time was recorded. The coagulation time of the blank group was limited to 7–8 min by adjusting the amount of CaCl_2_. All materials and blood were pre-warmed to 37 ℃ for 10 min before the experiment.

Supplementary information, method 2

Cytotoxicity assay

A CCK-8 assay was used to evaluate the viability of L929 cells. Briefly, SEC-Fe@Ca was sterilized and immersed in the cell culture medium at concentrations of 0.5, 1, 2, and 4 mg/mL. After the mixture was incubated at 37 °C for 24 h, the supernatant was collected. SEC-Fe@Ca extract was used for the test. L929 cells were incubated for 24 h at 100% humidified atmosphere with 5% CO_2_ at 37 °C, and the extract was added to the L929 cells, which were then cultivated for 24, 48, and 72 h. The extract was replaced daily. All measurements were repeated five times. L929 cells treated with the culture medium served as the blank group. The CCK-8 assay was performed according to the manufacturer’s protocol.

Supplementary information, method 3

Hemolysis assay

Red blood cells were obtained after centrifugation (10 min, 1000 rpm) and diluted to a concentration of 5% using saline. The samples and RBC suspension were mixed (0.5, 1, 2 and 4 mg/mL) and then incubated at 37 °C for 15 min. The mixture was then centrifuged at 3000 rpm for 10 min to separate the supernatant, and the absorbance of the supernatant was measured at 540 nm to evaluate hemolysis ratio. PBS served as the negative control and deionized water served as the positive control. All measurements were repeated at least three times.

$$Hemolysis ratio \left( \% \right)={(OD}_{S}-{OD}_{n})/{(OD}_{p}-{OD}_{n})\times100\%$$

${OD}_{S}$, ${OD}_{n}$and ${OD}_{p}$ are the absorbance values of the samples, negative control, and positive control, respectively.

Supplementary information, method 4

Long-term toxicity to the main organs after hemostatic treatment in vivo

New Zealand white rabbits were used as the animal model to evaluate the long-term toxicity of SEC-Fe@Ca to main organs. After successful hemostasis in the femoral artery model, the rabbits were raised normally for 1, 7, and 14 days. On days 1, 7, and 14, five rabbits were sacrificed, and the main organs including the heart, liver, spleen, lung and kidney were collected for tissue histology analysis to determine the long-term toxicity of SEC-Fe@Ca.


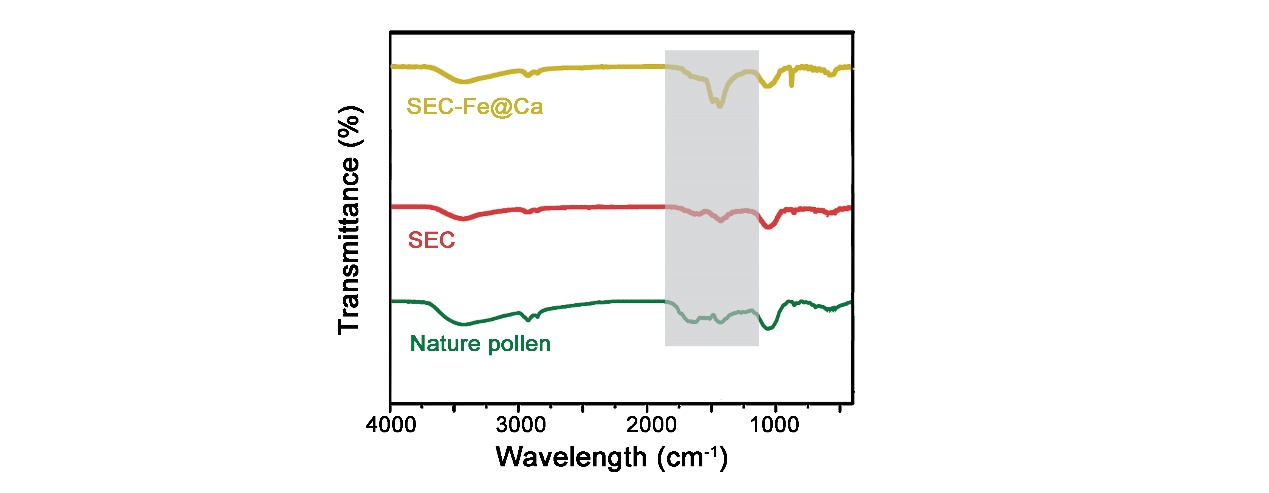


**Figure. S1** Full Fourier transform-infrared spectra of nature pollen, SEC, and SEC-Fe@Ca.


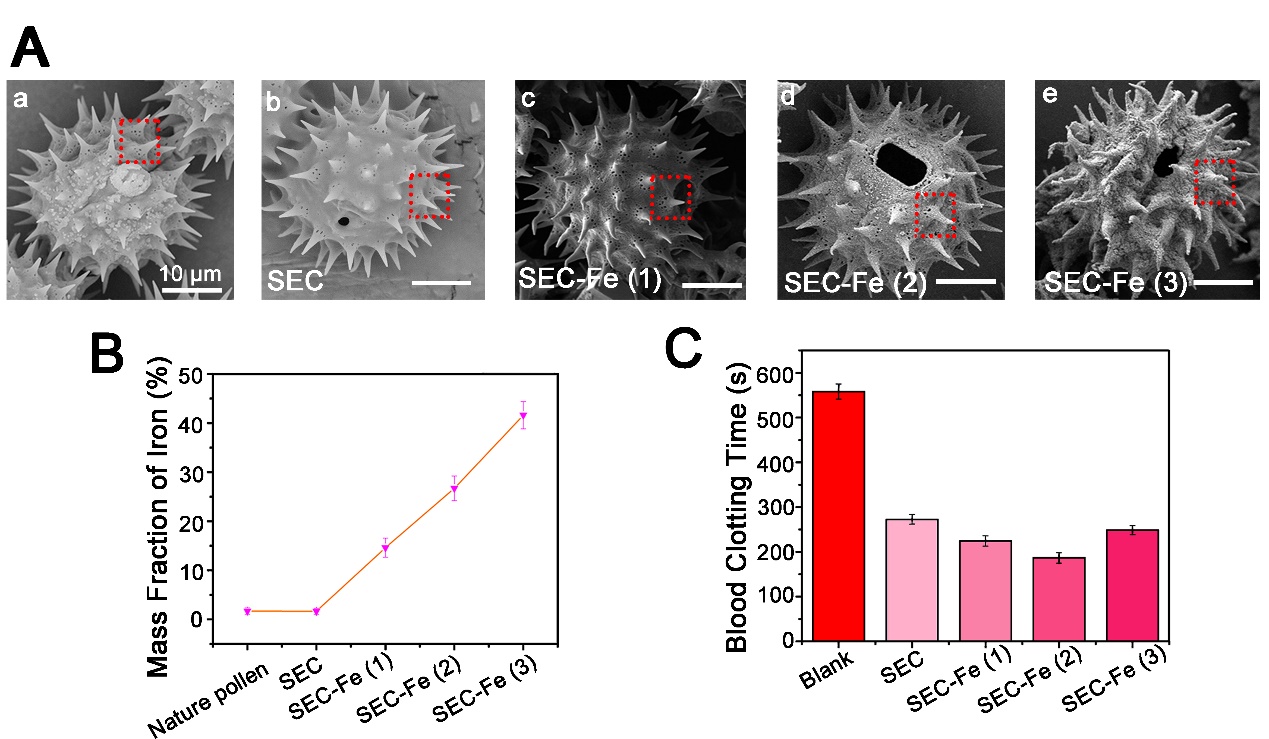


**Figure S2.** (A) Scanning electron microscopy (SEM) images of natural sunflower pollen, SEC, and SEC-Fe with different Fe_3_O_4_ loadings. (B) Mass fraction of iron in different samples. (C) In vivo blood clotting time of different samples.


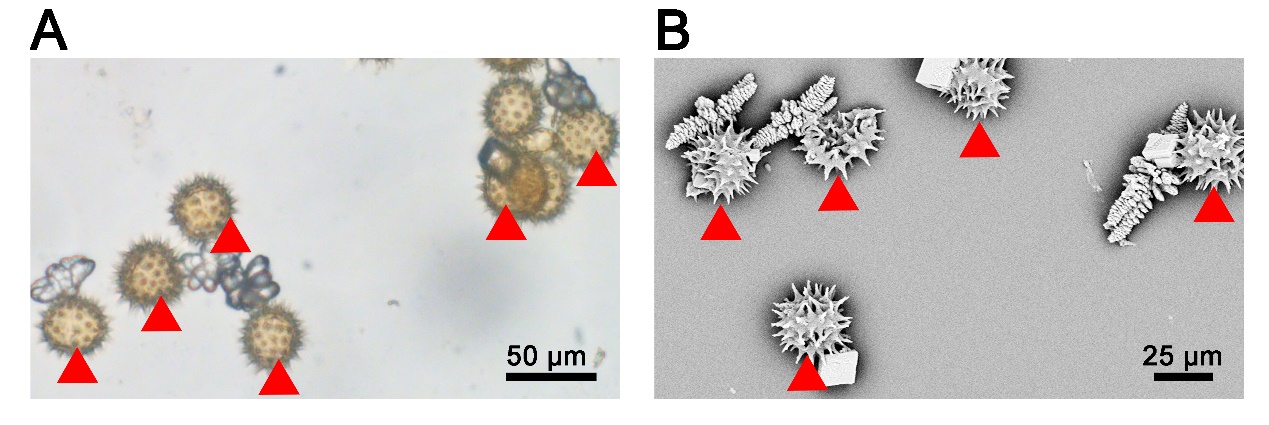


**Figure S3.** Optical microscope (A) and scanning electron microscopy (B) images of Janus SEC-Fe@Ca.


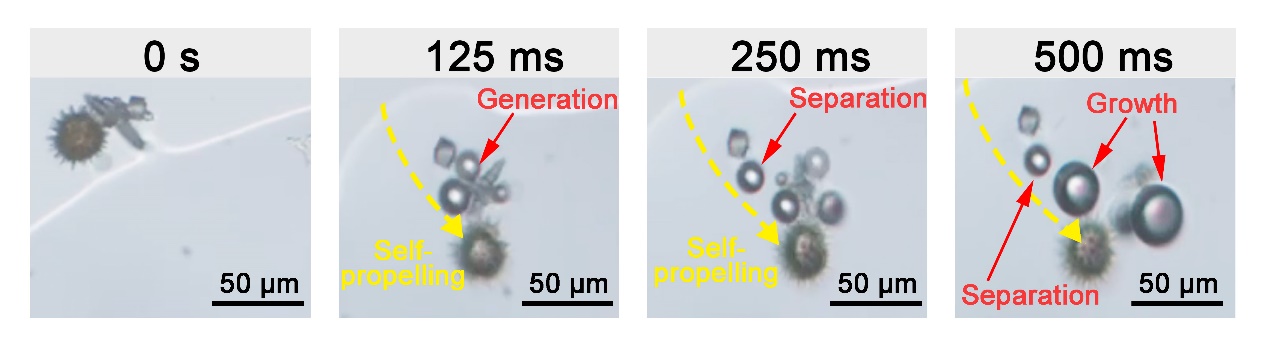


**Figure S4.** Motion behavior of self-propelling SEC-Fe@Ca in acid situation.

**
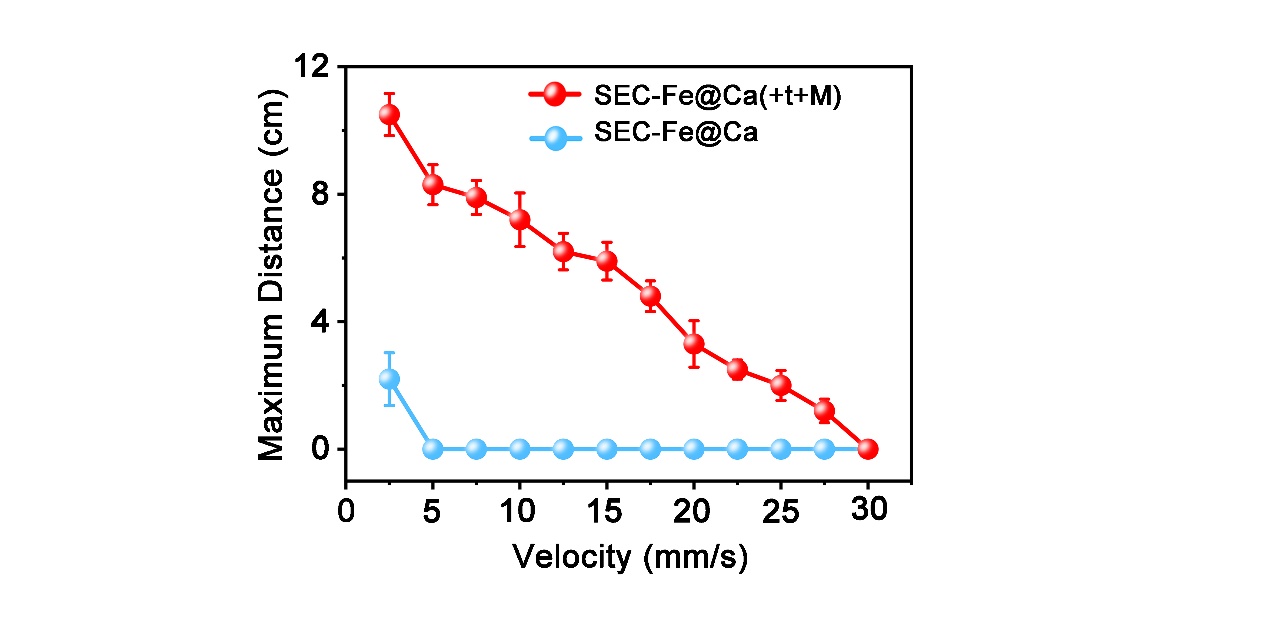
**

**Figure S5.** Maximum upstream distance of SEC-Fe@Ca and SEC-Fe@Ca(+t+M) in water flowing at different flow rates at 0° feeding angles.


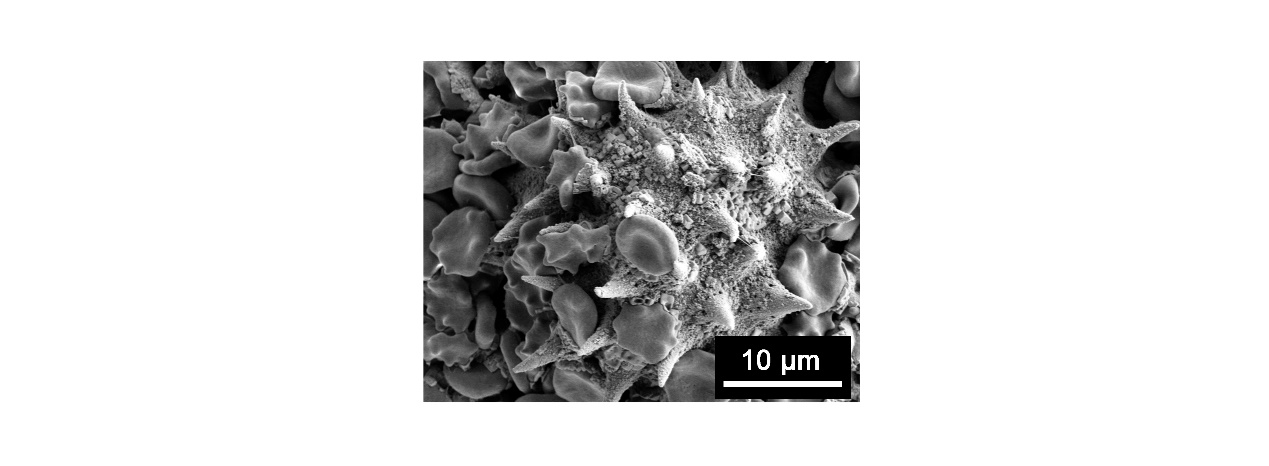


**Figure S6.** Scanning electron microscopy image of echinocytes treated with SEC-Fe@Ca(+t+M).


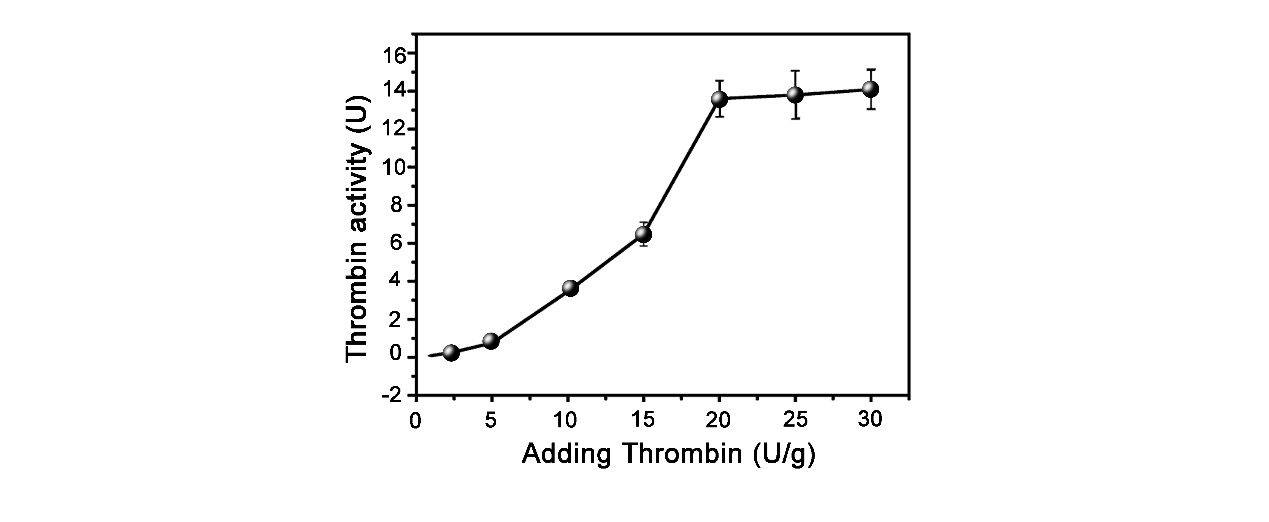


**Figure S7.** Thrombin activity as a function of loaded thrombin onto SEC-Fe@Ca.


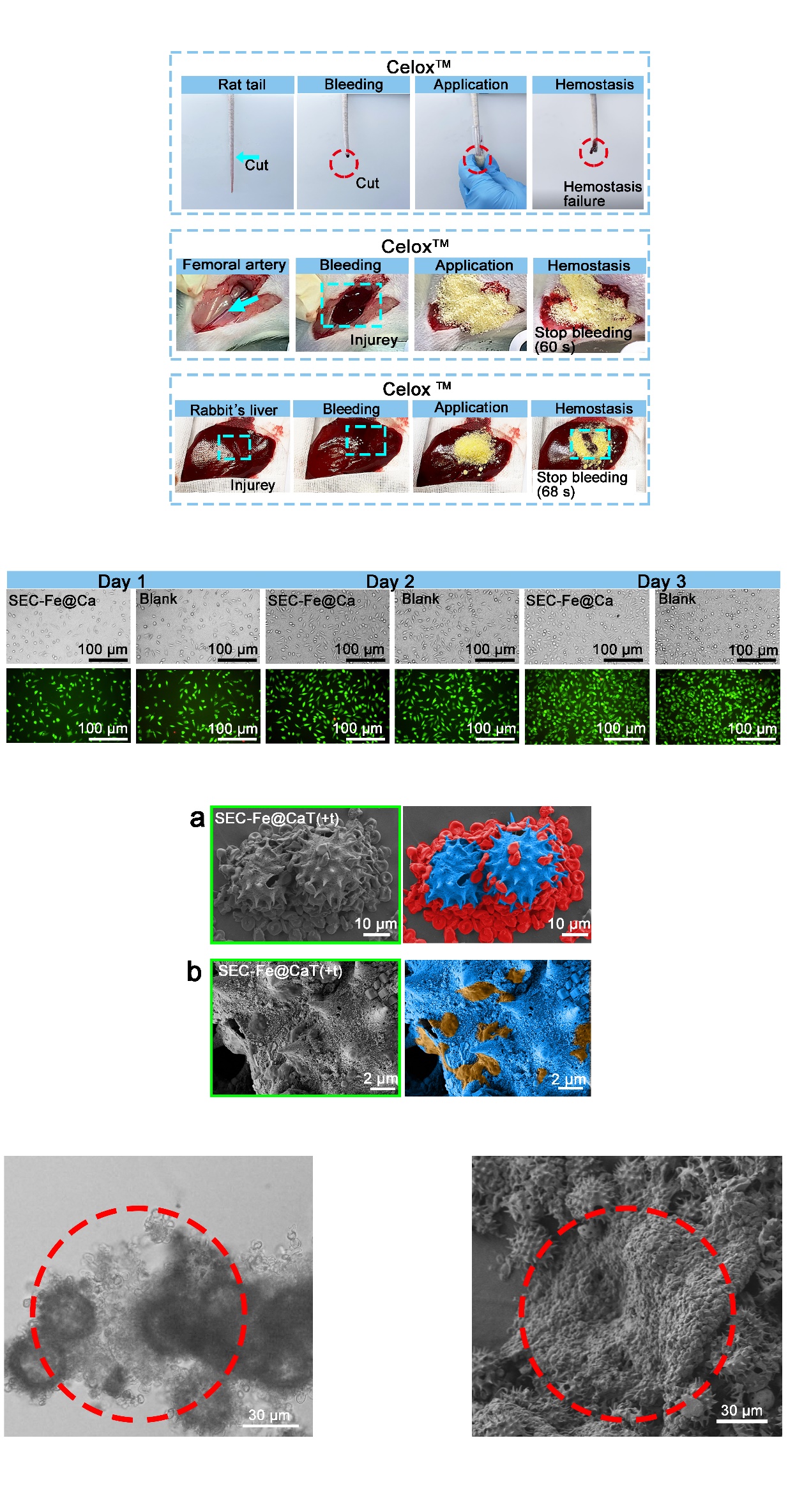


**Figure S8.** Pseudocolor scanning electron microscopy images of (A) red blood cells and (B) platelets treated with SEC-Fe@CaT(+t) and scanning electron microscopy images. Red pseudocolor represents erythrocytes, brown pseudocolor represents platelets, and blue pseudocolor represents SEC-Fe@CaCO_3_ particle.


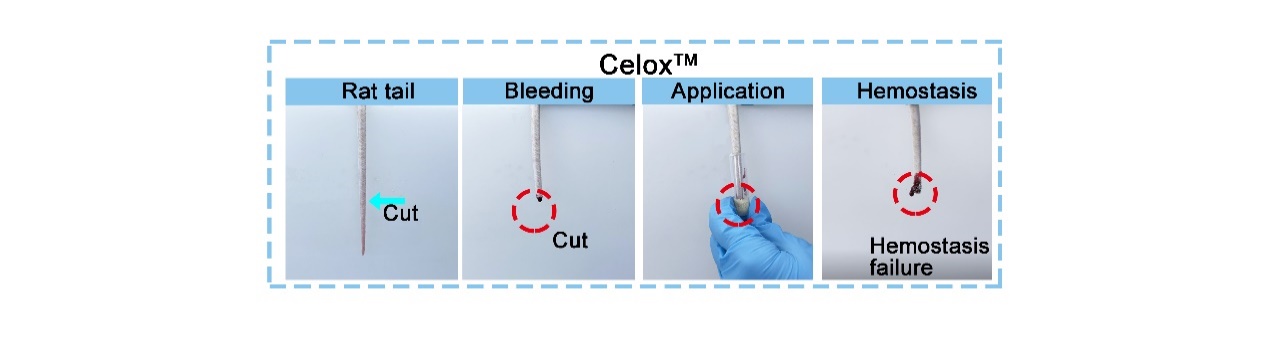


**Figure S9.** Hemorrhage control processes of Celox^TM^ on rat tail.


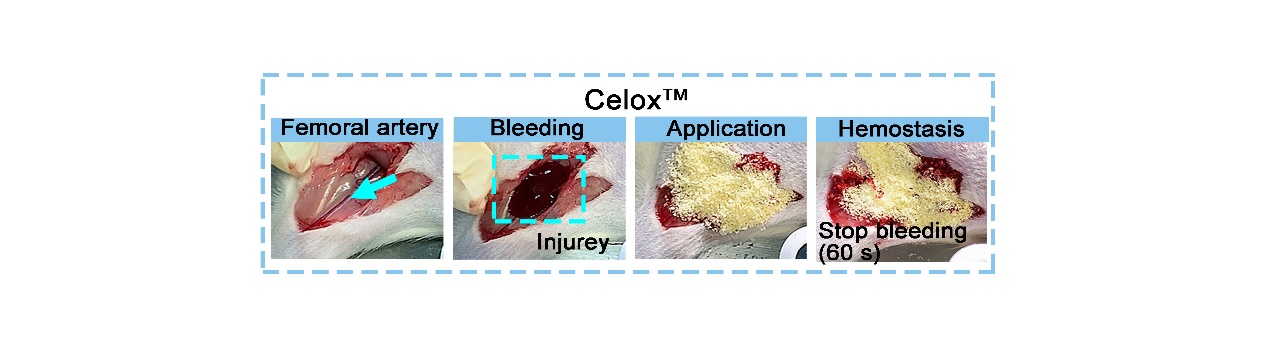


**Figure S10.** In vivo hemorrhage control processes of Celox^TM^ on femoral artery.


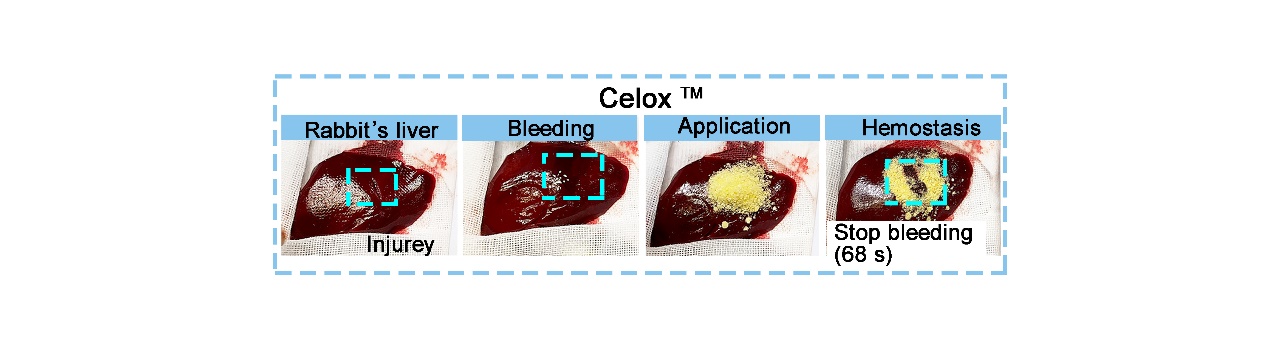


**Figure S11.** In vivo hemorrhage control processes of Celox^TM^ on liver.


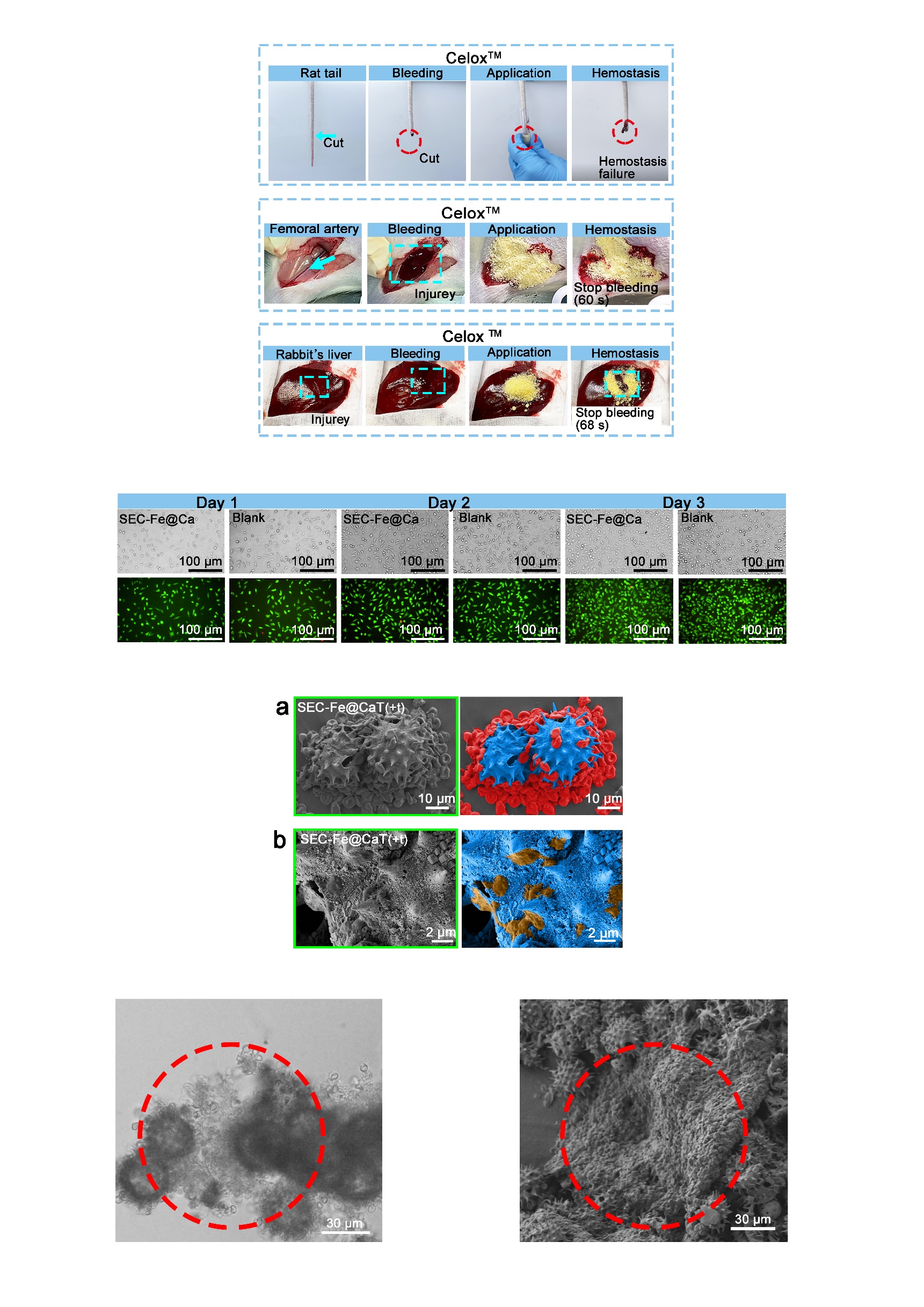


**Figure S12.** Fluorescence and bright field microscopy images of L929 cell cultivated with SEC-Fe@Ca for 1, 2, and 3 days.


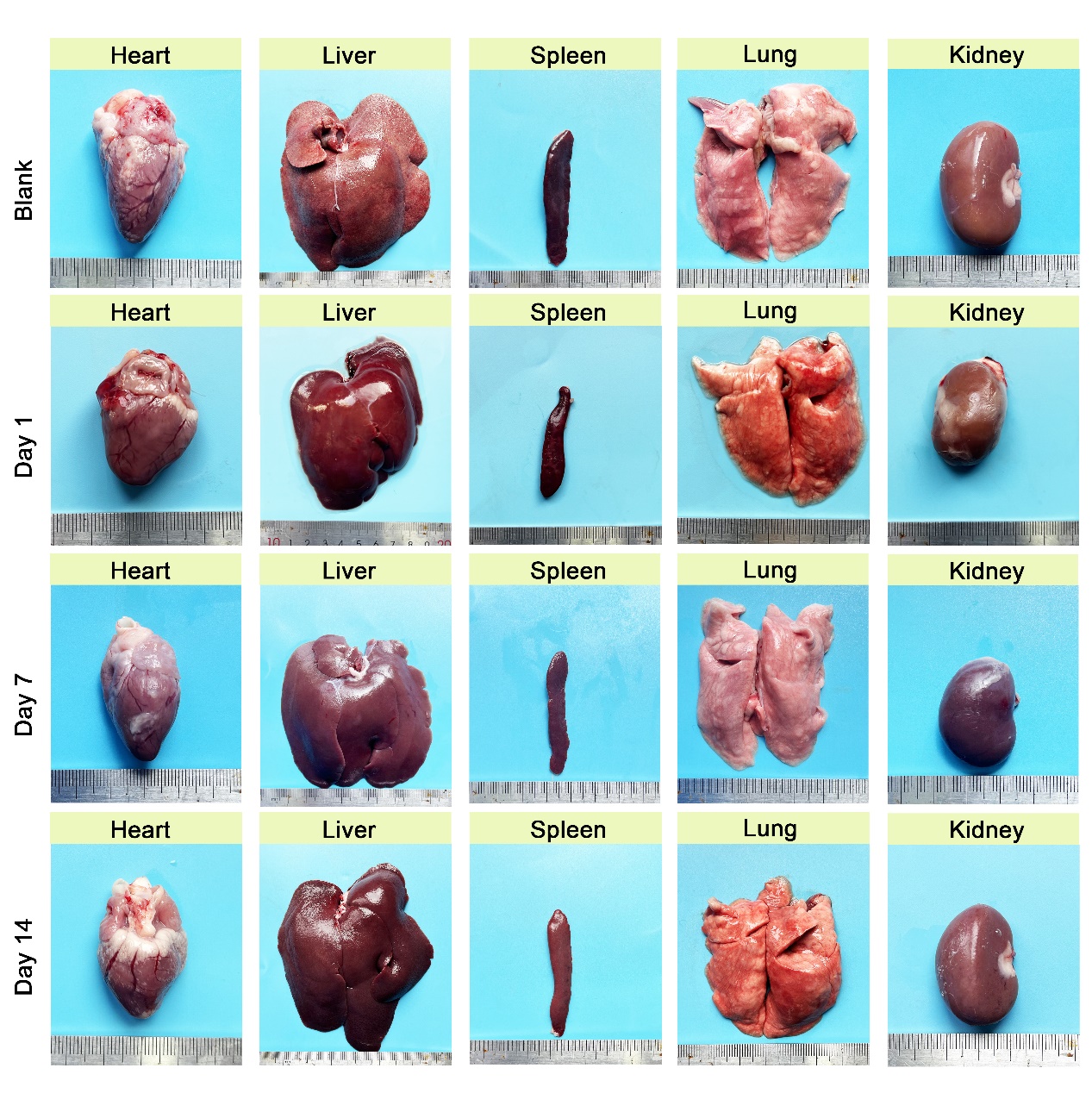


**Figure S13.** Various organs from New Zealand white rabbit treated with SEC-Fe@CaT.


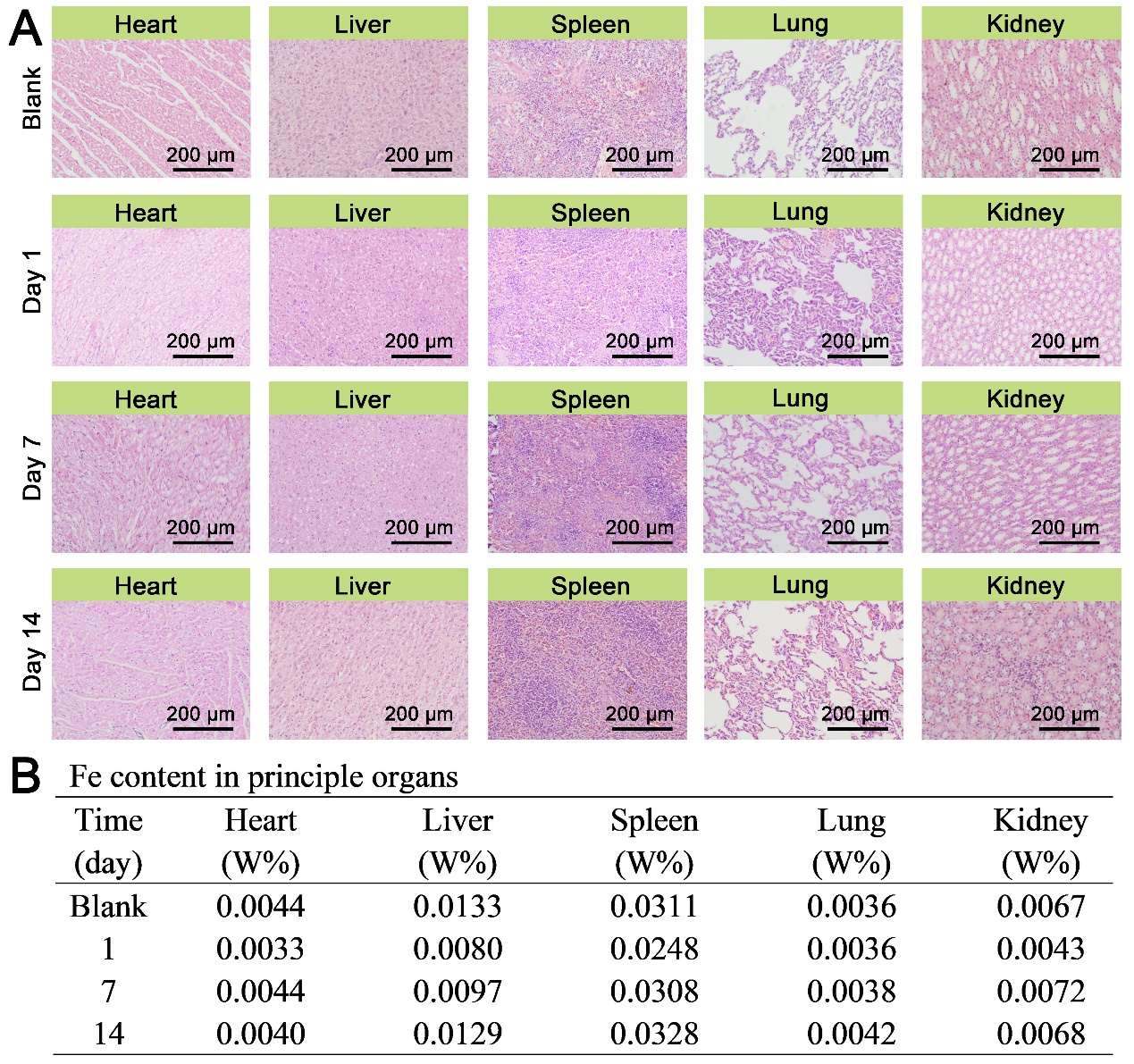


**Figure S14.** (A) Tissue section from main organs of rabbit after hemostatic treatment with SEC-Fe@CaT. (B) Concentration of iron ion in the main organs after hemostatic treatment of SEC-Fe@CaT for 1, 7, and 14 days.
